# Creatine in the fetal brain: A regional investigation of acute global hypoxia and creatine supplementation in a translational fetal sheep model

**DOI:** 10.3389/fncel.2023.1154772

**Published:** 2023-03-30

**Authors:** Nhi T. Tran, Anna M. Muccini, Nadia Hale, Mary Tolcos, Rod J. Snow, David W. Walker, Stacey J. Ellery

**Affiliations:** ^1^The Ritchie Centre, Hudson Institute of Medical Research, Melbourne, VIC, Australia; ^2^Department of Obstetrics and Gynaecology, Monash University, Melbourne, VIC, Australia; ^3^School of Health and Biomedical Sciences, RMIT University, Melbourne, VIC, Australia; ^4^Institute for Physical Activity and Nutrition, Deakin University, Melbourne, VIC, Australia

**Keywords:** hypoxia-ischemia encephalopathy, creatine metabolism, neuroprotection, perinatal asphyxia (PNA), perinatal brain injury

## Abstract

**Background:**

Creatine supplementation during pregnancy is a promising prophylactic treatment for perinatal hypoxic brain injury. Previously, in near-term sheep we have shown that fetal creatine supplementation reduces cerebral metabolic and oxidative stress induced by acute global hypoxia. This study investigated the effects of acute hypoxia with or without fetal creatine supplementation on neuropathology in multiple brain regions.

**Methods:**

Near-term fetal sheep were administered continuous intravenous infusion of either creatine (6 mg kg^–1^ h^–1^) or isovolumetric saline from 122 to 134 days gestational age (dGA; term is approx. 145 dGA). At 131 dGA, global hypoxia was induced by a 10 min umbilical cord occlusion (UCO). Fetuses were then recovered for 72 h at which time (134 dGA) cerebral tissue was collected for either RT-qPCR or immunohistochemistry analyses.

**Results:**

UCO resulted in mild injury to the cortical gray matter, thalamus and hippocampus, with increased cell death and astrogliosis and downregulation of genes involved in regulating injury responses, vasculature development and mitochondrial integrity. Creatine supplementation reduced astrogliosis within the corpus callosum but did not ameliorate any other gene expression or histopathological changes induced by hypoxia. Of importance, effects of creatine supplementation on gene expression irrespective of hypoxia, including increased expression of anti-apoptotic (*BCL-2*) and pro-inflammatory (e.g., *MPO, TNFa, IL-6, IL-1*β) genes, particularly in the gray matter, hippocampus, and striatum were identified. Creatine treatment also effected oligodendrocyte maturation and myelination in white matter regions.

**Conclusion:**

While supplementation did not rescue mild neuropathology caused by UCO, creatine did result in gene expression changes that may influence *in utero* cerebral development.

## 1. Background

Creatine is an amino acid derivate and its phosphorylated form, phosphocreatine, is a compound with the important role in supporting high-energy metabolism through the spatial and temporal buffering of cellular ATP during periods of high metabolic demand (Wyss and Kaddurah-Daouk, 2000). An increase of creatine by exogenous administration can result in an increase in tissue total creatine content (creatine plus phosphocreatine), allowing for a greater and more prolonged provision of phosphocreatine within cells (Wallimann et al., 2011). This has the effect of limiting cellular energy failure and sustaining redox homeostasis during periods of oxygen deprivation (Andres et al., 2005; Shen and Goldberg, 2012). Accordingly, dietary creatine supplementation is proposed as a prophylactic treatment strategy for pathologies that involve hypoxia-mediated energy failure such as perinatal hypoxic-ischemic encephalopathy (HIE) (Ellery et al., 2016).

The near-term fetal brain is particularly vulnerable to hypoxic-ischemic (HI) injury with even mild HIE events associated with neurodevelopmental deficits and delays (Hayes et al., 2018). A complex interplay between tissue oxygenation, cellular and mitochondrial function, and the generation of neurotoxic cascades leads to increased cell death, neuro-inflammation, oxidative stress (Gunn and Bennet, 2009), and ultimately, the clinical manifestation of HIE (Vannucci, 2000; Perlman, 2007). Targeting one or some of these mechanisms may be beneficial in reducing or even preventing the development of HIE. Of note, near-term HIE often presents as injury to the cerebral cortex, hippocampus, thalamus, and basal ganglia (striatum) (Barkovich et al., 1995). This increased injury susceptibility is attributable to the high metabolic needs of these particular brain regions (Martin et al., 1997; Sie et al., 2000; Okereafor et al., 2008).

Increasing the extracellular and intracellular availability of creatine and thus phosphocreatine to support cerebral metabolism within vulnerable brain regions may thereby attenuate the severity of HIE. Indeed, previous preclinical investigations have documented the neuroprotective effects of creatine following perinatal hypoxic events, having demonstrated reduced cerebral cell death and the size of infarcts, and improved behavioral function (Tran et al., 2021a). We have previously reported in near-term fetal sheep that higher circulating creatine levels were associated with reduced degrees of hypoxemia, reduced efflux of cerebral extracellular reactive oxygen species (ROS), and reduced extracellular lactate and pyruvate following global hypoxia induced by umbilical cord occlusion (UCO) (Tran et al., 2022). Despite indications of improved cerebral metabolism during the phase of secondary energy failure, we found in the same fetal sheep cohort that creatine supplementation did not reduce the dysregulation of mitochondrial respiration within hippocampal and white matter tissue samples, when studied *in vitro* at 72 h after the UCO insult (Muccini et al., 2022). This suggests that fetal creatine supplementation may be neuroprotective *via* other mechanisms. Indeed, a range of studies encompassing juvenile through to adult brain analyses suggest creatine has anti-inflammatory and antioxidant effects and can support cerebral vasculature perfusion (Prass et al., 2006; Beal, 2011; Riesberg et al., 2016; Sestili et al., 2016), see review (Muccini et al., 2021). While there is much promise that creatine might reduce the cellular pathology following HI, these neuroprotective outcomes have yet to be demonstrated in the near-term fetal brain.

Using the same cohort of fetal sheep where we previously demonstrated regional creatine loading in the fetal brain following direct intravenous infusion (Tran et al., 2021b,^2022^; Muccini et al., 2022), the aim of the present study was to identify the neuropathological effects induced by mild UCO within brain regions typically affected by hypoxia. We then assessed if fetal creatine supplementation could ameliorate such hypoxia-induced effects. To do this, we assessed changes in mRNA expression of multiple genes relating to key biological processes and pathological mechanisms involved in HI. We also investigated the effects on the key cell populations within the brain (neurons, oligodendrocytes, microglia, astrocytes, blood vessels) using immunohistochemical markers.

The second aim was to assess the effects of creatine itself on the fetal brain, irrespective of hypoxia. The brain possesses both a capacity to load creatine but also an inherent capacity to synthesis creatine (Braissant et al., 2007; Béard and Braissant, 2010). While it is not yet clear at what point in gestation the human fetus is able to independently synthesize creatine *de novo*, and in the brain in particular, it seems that the fetal requirements of creatine depend heavily on maternal creatine levels (Bianchi et al., 2000; Dickinson et al., 2014). Interestingly, studies have indicated that maternal creatine supplementation influences the morphological and functional development of neurons in rodent offspring, enhancing overall neuronal excitability and improving long-term potentiation (Sartini et al., 2016, 2019). Other than these studies by Sartini et al. (2016, 2019) there is limited data on the effects of increased creatine levels within the immature brain. Investigating the mechanisms by which creatine supplementation could alter basal biological processes in the fetal brain is important for establishing its safety profile for use in pregnancy and during the perinatal period.

## 2. Materials and methods

### 2.1. Experimental procedures

This study utilized post-mortem fetal brain tissue collected from previously published studies detailing the experimental model and physiological outcomes (Tran et al., 2021b,^2022^). The use of animals was approved by Monash Medical Centre Animal Ethics Committee (MMCA-2017-02) and was conducted in accordance with the National Health and Medical Research Council Code of Practice for the Care and Use of Animals for Scientific Purposes (Eighth Edition).

Briefly, 28 time-mated pregnant Border–Leicester/Merino cross ewes carrying singleton fetuses at 118 days’ gestational age (dGA; term is ∼145–147 dGA) were placed under general anesthesia using 2% isoflurane in oxygen. Under strict sterile conditions, surgery involved exposure of the fetal head and forelimbs *via* a midline maternal abdominal incision and approximately a 10 cm incision of the uterus. The fetal left brachial artery and vein were catheterized for blood sampling and creatine or saline infusion, respectively. A microdialysis probe was also inserted into the right cerebral hemisphere as detailed elsewhere (Tran et al., 2022). The fetus was then withdrawn further from the uterus and an inflatable silastic vascular occluder (type-OC 16 mm, *in vivo* Metric, Healdsburg, CA, USA) was placed around the umbilical cord and anchored to the fetal abdomen. The fetus was then returned to the uterus, and all fetal, uterine and maternal incisions were repaired, and catheters exteriorized *via* a small (1–1.5 cm) incision in the maternal right flank. Ewes were recovered from surgery and placed in individual pens and in the company of other sheep at the Monash Medical Centre animal house holding facility, maintained on a 12 h light/dark cycle (8:00 a.m.–8:00 p.m.) at an ambient temperature of ∼20^°^C. Ewes were fed twice daily with a lucerne chaff mixture and had access to water *ab libitum*. The overall wellbeing of each ewe, and food and water intake was monitored daily throughout the entire experiment.

Fetuses were randomly assigned to one of the following four treatment groups: sham UCO with saline (*n* = 6; SalCon) or creatine infusion (*n* = 7; CrCon); or UCO for 10 min with saline (*n* = 8; SalUCO) or creatine infusion (*n* = 7; CrUCO). All creatine-infused fetuses were given a continuous intravenous infusion of creatine from 121 dGA at 6 mg kg^–1^.h^–1^ (18 mg h^–1^, based on the historical observation of an average fetal weight of ∼3 kg) diluted in saline and delivered at 1.5 ml h^–1^ and continued until the end of the experiment at 134 dGA when post-mortem was performed. Saline-infused fetuses received an isovolumetric administration of 0.9% NaCl, pH 7.4 for the same duration. Mild global fetal hypoxia was induced transiently at 131 dGA where the umbilical cord was completely occluded (UCO) for 10 min, followed by recovery *in utero* for 72 h until post-mortem. At post-mortem, the ewe and fetus were humanely killed using an overdose of pentobarbitone (100 mg kg^–1^; Lethabarb; Virbac, Milperra, NSW, Australia) injected intravenously to the ewe, as previously described (Tran et al., 2021b). The fetal brain was rapidly removed from the skull by cutting the cranial nerves, pituitary stalk, and the cervical spinal cord, and then divided into right and left hemispheres along the midline. Samples of cortical gray matter (GM), white matter (WM; combined sampling from the subcortical and periventricular white matter), hippocampus, striatum (combined sampling from the putamen and caudate nucleus), and thalamus were dissected from the left hemisphere and snap frozen in liquid nitrogen and stored at −80^°^C until analysis of mRNA expression. The right hemisphere was left intact and fixed by immersion in 4% paraformaldehyde (0.1 M; pH 7.4) for 72 h and then cut coronally into 5 mm-thick rostro-caudal blocks (∼10 blocks per brain) and embedded in paraffin.

### 2.2. RT-qPCR

Total RNA extraction and cDNA preparation was conducted as previously described (Muccini et al., 2022). Briefly, RNA was extracted from frozen brain tissue (60–80 mg) using an RNA Mini Kit (PureLink RNA Mini Kite, Thermofisher, Melbourne, VIC, Australia) following the manufacturer’s instructions. RNA yield was determined by spectrophotometry (Nanodrop, Analytical Technologies, Biolab, Concord, ON, Canada). cDNA was transcribed from RNA, then pre-amplified at 25 ng μl^–1^ with a mixture of pooled 20X TaqMan gene expression assay probes (Life Technologies, ThermoFisher, USA), Taqman PreAmp Master Mix (2X; Applied Biosystems; Thermo Fisher Scientific, Lithuania).

Genes involved in cellular regulation, inflammation, metabolism, oxidative stress and vasculature development are listed in Table 1. Expression of these genes was analysed across all of the flash-frozen brain regions. Genes associated with mitochondrial integrity, cell death and metabolic pathways (Table 1) were also assessed in the striatum and thalamus, adding to previous data published on the gray matter, white matter and hippocampus by Muccini et al. (2022). Gene expression was analysed using a Fluidigm Dynamic array Biomark HD system (Fluidigm, USA). Gene expression was determined as relative expression calculated by change in cycle threshold (ΔCt) between Ct of each gene of interest and the geometric average of three endogenous housekeeping genes, *RPL32*, *RPS16*, and *OAZ1*. Levels of mRNA expression relative to geometrical average of house-keeping genes were determined using the 2^–ΔΔCT^ method (Livak and Schmittgen, 2001). Due to technical issues, frozen samples from one animal in each of the SalCon and CrUCO groups could not be analysed; thus, the revised group numbers for RT-qPCR analysis are, SalCon, *n* = 5; CrUCO, *n* = 6.

**TABLE 1:** Genes of interest.

Biological process	Gene name	ID	Taqman code
Cellular regulation	Hypoxia-inducible factor alpha*	*HIF1-*α	Oa04877334_m1
	Transforming growth factor-beta	*TGF-*β	Oa04259484_m1
	Nuclear factor kappa B subunit 1	*NF*κ*B1*	Oa04837805_m1
	Neuregulin 1	*NRG1*	Oa04912037_m1
	Regulator of calcineurin 1	*RCAN1*	Oa04658608_m1
	Collagen type I alpha 1 chain	*COL1A1*	Oa01463861_gH
	Collagen type III alpha 1 chain	*COL3A1*	Oa04910910_m1
	Peroxisome proliferator-activated receptor gamma	*PPAR*γ	Oa01208835_m1
Cell death	Caspase 3*	*CAS3*	Oa04817361_m1
	BCL2 associated X*	*BAX*	Oa03211776_g1
	BCL2 antagonist/killer 1*	*BAK1*	Oa04906761_m1
	BCL2*	*BCL-2*	Oa04888158_m1
	Beclin 1*	*BECN1*	Oa01011595_g1
Inflammation	C-C motif chemokine 2	*CCL2*	Oa04677078_m1
	Tumor necrosis factor alpha	*TNFa*	Oa04655425_g1
	Interleukin 1 beta	*IL-1*β	Oa04656322_m1
	Interleukin 6	*IL-6*	Oa04656315_m1
	C-X-C Motif chemokine ligand 8 [interleukin 8 (IL-8)]	*CXCL8*	Bt03211906_m1
	Toll-like receptor 3	*TLR3*	Oa04657622_m1
	Toll-like receptor 4	*TLR4*	Oa04656419_m1
	Cyclooxygenase 1	*PTGS1*	Oa04658906_m1
	Cyclooxygenase 2	*PTGS2*	Oa04657348_g1
Metabolic pathways	Glucose Transporter 1	*SLC2A5*	Oa04659413_g1
	Solute carrier family 6 member 8*	*SLC6A8*	Oa04855795_g1
	Choline transporter	*SLC5A7*	Oa03234527_m1
	Estrogen-related receptor alpha*	*ERR*α	Oa04890968_m1
	Creatine kinase, mitochondrial*	*CKMT*	Oa04870503_m1
	Fatty acid synthase	*FASN*	Oa04857875_g1
	Pyruvate dehydrogenase kinase 1	*PDK1*	Oa04668108_m1
	Protein kinase AMP-activated catalytic subunit alpha 1*	*PRKAA1*	Oa03276076_m1
	Protein kinase AMP-activated catalytic subunit alpha 2*	*PRKAA2*	Oa04283525_m1
Mitochondrial integrity	Mitochondrial fusion protein 1*	*MFN1*	Oa04295229_m1
	Mitochondrial fusion protein 2*	*MFN2*	Oa04843335_g1
	Fission protein 1 *	*FIS1*	Oa03227775_m1
	Dynamin 1 like*	*DNM1L*	Oa04916359_m1
	Optic atrophy 1*	*OPA1*	Oa04313744_m1
	Mitochondrial fission factor*	*MFF*	Oa03220969_g1
	Beta-2-microglobulin*	*B2M*	Oa04900279_mH
	tRNA methyltransferase 11 homolog*	*TRMT11*	Oa03251454_g1
	Succinate-quinone oxidoreductase – Complex II*	*NDUFBB* (CII)	Oa04659352_g1
	Cytochrome bc1 – Complex III*	*UQCRH* (CIII)	Oa04655907_g1
	Cytochrome c oxidase – Complex IV*	*LOC101105* (CIV)	Oa04838042_g1
	ATP synthase – Complex V*	*ATP6PO* (CV)	Oa04655945_m1
	Mitochondria elongation factor 1*	*MIEF1*	Oa04733603_m1
	Cardiolipin synthase 1*	*CRLS1*	Oa04750955_g1
	Mitochondrial transcription factor A*	*TFAM*	Oa03260079_g1
	Myostatin*	*MSTN*	Oa04654279_m1
	SIRT1*	*SIRT1*	Oa04313950_m1
	SIRT3*	*SIRT3*	Oa04666037_m1
	Carnitine palmitoyl transferase 1A*	*CPT1A*	Oa04658023_m1
Oxidative stress	Myeloperoxidase	*MPO*	Oa04654413_g1
	NADPH oxidase 1	*NOX1*	Oa04709255_g1
	NADPH oxidase 2 (NOX2)	*CYBB*	Oa04793417_m1
	Glutathione peroxidase 1	*GPX1*	Oa04911462_g1
	Catalase	*CAT*	Oa03228713_m1
	Superoxide dismutase 2, mitochondrial	*SOD2*	Oa04657474_m1
	Superoxide dismutase 2, extracellular	*SOD3*	Oa04858164_m1
	Nitric oxide synthase 1	*NOS1*	Oa04714367_m1
	Nitric oxide synthase 2	*NOS2*	Oa04876175_m1
	Nitric oxide synthase 3	*NOS3*	Oa04907031_mH
	Glutathione-disulfide reductase	*GSR*	Oa04903941_m1
	Nuclear factor erythroid 2-related factor 2	*NFE2L2*	Oa04815261_g1
Vasculature development	Vascular endothelial growth factor A	*VEGFA*	Oa04653812_m1
	Occludin	*OCLN*	Oa04728972_m1
	Angiopoietin 2	*ANGPT2*	Oa04857533_m1
	Meis homeobox 1	*MEIS1*	Oa04914453_m1
	Claudin-1	*CLDN1*	Oa03217991_m1
Housekeeping genes	Ribosomal protein L32	*RPL32*	Oa04893129_g1
	Ribosomal protein S16	*RPS16*	Oa03225223_m1
	Ornithine decarboxylase antizyme 1	*OAZ1*	Oa03220471_g1

*Indicates genes assessed only within the striatum and thalamus.

### 2.3. Immunohistochemistry

Paraffin-embedded blocks were sectioned coronally at 8 μm thickness using a microtome across the parietal-temporal lobe and tissue sections placed on glass slides. For each animal, a total of two slides, corresponding to section 720 and section 1,120 according to the Michigan State University Sheep Atlas [Cx4 (rostral) and Cx7 (caudal); Supplementary Figure 1] were utilized per immunohistochemical marker. Slides were dewaxed in xylene and rehydrated in decreasing concentrations of ethanol. Slides were then washed in phosphate buffered saline (PBS; pH 7.4) and prepared for individual protocols for histochemical staining.

Details of specific immunohistochemistry protocols are provided in Supplementary Table 1. After antigen retrieval was conducted, slides were rinsed with PBS and blocked for endogenous peroxidases and then blocked for non-specific binding. Slides were then incubated overnight at 4^°^C in the presence of one of the following primary antibodies: mouse anti-NeuN (1:500; Millipore, Germany; CAT#: MAB377) for mature neurons; rabbit anti-ionized calcium binding adaptor molecule 1 (IBA-1; 1:1000; Wako Chemicals, USA; CAT#: 019-19741) for microglia; rabbit anti-glial fibrillary acidic protein (GFAP; 1:500; Dako, USA; CAT#: Z033401-2) for astrocytes; mouse anti-CNPase (1:500; Sigma-Aldrich, USA; CAT#: C5922) for pre-myelinating and myelinating oligodendrocytes and myelin; rabbit anti-myelin basic protein (MBP; 1:200; Chemicon International, USA; CAT# AB5864) for mature oligodendrocytes and myelin; and rabbit anti-Olig-2 (1:500; Millipore, Germany; CAT#: MABN50) for the entire oligodendrocyte lineage; rabbit anti-sheep serum (1:700; Sigma-Aldrich, USA; CAT#: S4265) for serum protein extravasation to detect blood brain barrier (BBB) permeability. Slides were then washed in PBS and incubated in secondary biotinylated IgG antibody raised in corresponding species (1:200). The slides were washed again and incubated with avidin-biotin complex (ABC Elite kit; 1:1:200 in PBS; Vectastain^®^, Vector Laboratories, UK) then visualized with 3,3’-diamniobenzidine solution (DAB; 1 tablet in 10 ml dH_2_O; MP Biomedicals, Australia). In addition, to assess cell death, a “Terminal deoxynucleotidyl transferase dUTP nick end labelling” (TUNEL) assay (ApopTag^®^ Peroxidase *in situ* Apoptosis Detection Kit, Millipore, USA) was conducted according to manufacturer’s instructions. All slides were then dehydrated and then cover-slipped using DPX (Merck, Germany). IBA-1 and Olig-2 immunohistochemical stains were counterstained with hematoxylin. Negative control sections where the primary antibody was omitted, were conducted for each staining protocol. No positive cellular staining and minimal background staining was observed for all these negative control sections.

### 2.4. Immunohistochemistry quantification

All analyses were undertaken by investigators blinded to the treatment groups. Slides were scanned at 20 × magnification using an Aperio Scanscope AT Turbo (Leica Biosystems, Germany). Regions of interest included the cortical grey matter (GM), subcortical white matter (SCWM), periventricular white matter (PVWM), corpus callosum (CC), caudate, putamen, internal and external capsule, thalamic nuclei and hippocampus [dorsal region: cornu ammonis (CA) 1–3 region and dentate gyrus (DG)] (Supplementary Figure 1). Regions of interest were outlined using Aperio Image Scope (Leica Biosystems, Germany). Six to ten fields of interest (FOI; 200 μm × 200 μm) were randomly placed within each brain region of interest but placed at similar locations for each animal; for smaller brain regions, i.e., hippocampus (dorsal region), FOI of 100 μm × 100 μm were used. For all analyses in which the same brain regions were present within both the rostral and caudal sections (GM, SCWM, PVWM, CC), statistical analysis (paired *t*-test) was undertaken to assess whether there was an effect of section sampling; if no sampling effect was found, analysis parameters were averaged for each animal. Only MBP-positive area coverage analysis within the SCWM revealed a section sampling difference and therefore results are presented separately (i.e., rostral and caudal).

#### 2.4.1. TUNEL analysis

For quantitative assessments of TUNEL-positive cell density, TUNEL-positive cells were manually counted in each FOI and then expressed as cells/mm^2^; means were calculated for each animal and a mean of means for each experimental group determined. Note that TUNEL data is only reported for the caudate, thalamus, CC and putamen adding to previous data published on the GM, WM and hippocampus (Muccini et al., 2022).

#### 2.4.2. Sheep serum analysis

Analysis of sheep serum extravasation from blood vessels into the surrounding parenchyma was used as an indicator of blood brain barrier (BBB) breakdown or compromise. FOI boxes were not used for sheep serum analysis, instead, whole brain regions of interest were outlined on each scanned section. Blood vessels were manually identified as either being intact: positively-stained serum limited to within blood vessel, or compromised: positively-stained serum within the parenchyma surrounding a blood vessel. A vessel with sheep serum extravasation was counted as one vessel regardless of the number of areas of serum leakage around a single connected blood vessel. Positively-stained serum without a visible blood vessel was not counted. The number of vessels exhibiting serum extravasation was counted for each brain region per fetus, and an average calculated for each experimental group.

#### 2.4.3. NeuN analysis

For assessment of NeuN-positive cell density in the GM, counts were conducted in four bins each roughly equivalent to cortical layer I (Bin 1), layer II and III (Bin 2), layer IV and V (Bin 3), and layer VI (Bin 4). If no differences were found between experimental groups for each bin, cell counts from all bins were summated and expressed as cell density (cells/mm^2^) within the entire GM sampling for each animal, and then an average calculated for each experimental group.

#### 2.4.4. IBA-1 and GFAP analysis

Changes to microglia and astrocytes that indicate a neuroinflammatory state were assessed using antibodies specific for microglia (IBA-1) and astrocytes (GFAP). IBA-1 and GFAP-immunoreactive cells were manually counted in each FOI and then an average calculated for each animal; the cell density (cells/mm^2^) for each was then determined. For quantitative assessments of IBA-1 and GFAP-positive area coverage to assess the proportion of the area occupied by IBA-1 and GFAP-positive cell bodies and branching processes, each FOI was exported, and the image processed using ImageJ software (version 2.0.0-rc-69/1.52p, National Institutes of Health). The “area coverage tool” in ImageJ was used to automatically quantify percentage of positive staining in each FOI; an average area coverage of multiple FOI was then calculated for each brain region in each fetus.

#### 2.4.5. Olig-2, CNPase, and MBP analysis

White matter development and integrity was assessed using oligodendrocyte lineage and myelination markers, e.g., Olig-2, a pan-oligodendrocyte marker for the entire oligodendrocyte lineage, CNPase for pre-myelinating and myelinating oligodendrocytes and myelin, and MBP for mature myelinating oligodendrocytes and myelin (see Figure 4). For Olig2-positive cell density in the GM, cell counts were conducted in Layer VI. As above, Olig-2- and MBP-positive cells were manually counted in each FOI and the cell density (cells/mm^2^) determined. For quantitative assessments of CNPase- and MBP-positive area coverage, an average area coverage (%) of multiple FOI was then calculated for each brain region in each fetus.

### 2.5. Statistical analysis

All statistical analyses and data visualization were conducted using GraphPad Prism (version 8.1.2; GraphPad Software, CA, United States). Data were assessed for normality using the Shapiro-Wilk Test with an alpha of 0.01 due to small sample sizes. RT-qPCR data expressed as relative change (2^–ΔΔCT^) were log-transformed to maintain normal distribution, and all statistical analysis and conducted on log transformed 2^–ΔΔCT^ data. For all analyses, data sets were assessed for main effects of UCO (*P*_*UCO*_), main effects of creatine treatment (*P*_*TREAT*_) and interactions between the main effects (*P*_*INT*_) by two-way ANOVA. Where a significant interaction was observed, post-hoc analysis was performed using Tukey’s multiple comparison test. RT-qPCR volcano plot data visualizations present the main effect of UCO, main effect of creatine, and post-hoc comparisons of SalUCO vs. CrUCO as fold changes. Data are presented as mean ± SD and *P* < 0.05 was considered statistically significant and a trend for significance was considered when *P* < 0.07.

## 3. Results

The systemic changes of blood gases, pH, cardiovascular responses, cerebral metabolism and oxidative responses to UCO and creatine supplementation have been reported elsewhere (Tran et al., 2022). Of note, all physiological and metabolic parameters had returned to pre-UCO levels by the time of the post-mortem, 72 h post-UCO.

### 3.1. Regional effects of global hypoxia and creatine supplementation on gene expression

**FIGURE 1:**
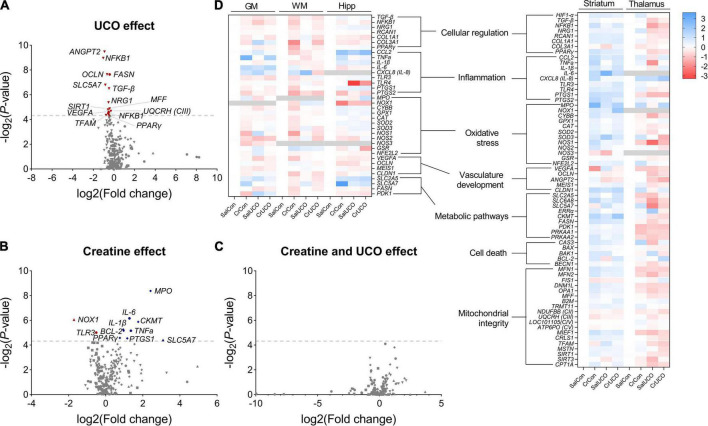
Volcano plot visualizations of **(A)** umbilical cord occlusion (UCO) and **(B)** creatine main effects, and **(C)** SalUCO vs. CrUCO post-hoc comparisons. Volcano plots show fold change [log_2_(fold change); i.e., log_2_(fold change = 2) = 1; x-axis] and all gene expression changes [−log_2_(adjusted *P*-value); y axis] in all brain regions: GM (●), WM (■), hippocampus (▲), striatum (◆) and thalamus (▼). Gray dashed line represents a *P*-value of 0.05 [−log_10_(0.05)≈4.139]. Names of genes with *P* < 0.05 are included. Genes that were downregulated are presented in red and those upregulated in blue. **(D)** Heat map representation of gene expression determined by reverse transcription-quantitative polymerase chain reaction (RT-qPCR) within saline control (SalCon; *n* = 5), creatine control (CrCon; *n* = 7), saline UCO (SalUCO; *n* = 7) and creatine UCO (CrUCO; *n* = 6) fetuses. Brain regions analysed include cortical grey matter (GM); white matter (WM); hippocampus (Hipp); striatum; and thalamus. Red indicates downregulation of mRNA expression; blue indicates upregulation relative to saline controls. Data are average log_2_ transformed fold change.

The effect of acute global hypoxia and creatine supplementation on genes of interest in the cortical GM, WM, hippocampus, striatum, and thalamus of the fetal sheep brain are summarized as volcano plots (Figures 1A–C) and a heat map (Figure 1D). Values are reported in Supplementary Table 2.

#### 3.1.1. Effect of UCO on regional gene expression

Umbilical cord occlusion resulted in significant downregulation of mRNA expression of multiple genes in the GM and thalamus (Figure 1A). Within the cortical GM, UCO resulted in downregulation of *NF*κ*B1* [*F*(1, 18) = 4.69, *P*_*UCO*_ = 0.044], *VEGFA* [*F*(1, 18) = 4.86, *P*_*UCO*_ = 0.041] and *FASN* expression [*F*(1, 18) = 10.23, *P*_*UCO*_ = 0.005]. These genes are involved in cellular regulation, vasculature growth and fatty acid synthesis, respectively (Figure 1D and Supplementary Table 2). In the thalamus, UCO resulted in decreased expression of cellular regulatory genes: *TGF-*β [*F*(1, 19) = 8.04, *P*_*UCO*_ = 0.011], *NF*κ*B1* [*F*(1, 17) = 13.23, *P*_*UCO*_ = 0.002], *NRG1* [*F*(1, 19) = 5.986, *P*_*UCO*_ = 0.024] and *PPAR*γ [*F*(1, 19) = 4.3, *P*_*UCO*_ = 0.050]; the choline transporter, *SLC5A7* [*F*(1, 17) = 8.62, *P*_*UCO*_ = 0.009]; genes involved in mitochondrial biogenesis *MFF* [*F*(1, 19) = 5.23, *P*_*UCO*_ = 0.034], *SIRT1* [*F*(1, 19) = 5.11, *P*_*UCO*_ = 0.036], and *TFAM* [*F*(1, 19) = 4.45, *P*_*UCO*_ = 0.049]; and the gene encoding mitochondrial complex III [*F*(1, 19) = 4.94, *P*_*UCO*_ = 0.039]. Expression of the angiogenic gene, *ANGPT2* [*F*(1, 19) = 13.98, *P*_*UCO*_ = 0.001] and tight-junction protein *OCLN* [*F*(1, 19) = 9.86, *P*_*UCO*_ = 0.005] were also downregulated with UCO in the thalamus (Figure 1D and Supplementary Table 2). No gene expression changes were found in the WM, hippocampus and striatum as a result of the UCO.

#### 3.1.2. Effect of creatine treatment on regional gene expression

Creatine treatment irrespective of hypoxia resulted in altered gene expression within the cortical GM, hippocampus and striatum (Figure 1B). Within the cortical GM, creatine treatment upregulated the pro-inflammatory genes *TNFa* [*F*(1, 12) = 6.28, *P*_*TREAT*_ = 0.028] and *IL-6* [*F*(1, 17) = 7.45, *P*_*TREAT*_ = 0.014], and downregulated *TLR3* expression [*F*(1, 18) = 5.52, *P*_*TREAT*_ = 0.031] (Figure 1D and Supplementary Table 2). Within the hippocampus, *NOX1* expression, a gene encoding NADPH oxidase 1, which has a primary function in generating free radicals, was downregulated with creatine treatment [*F*(1, 15) = 7.64, *P*_*TREAT*_ = 0.015]. Also within the hippocampus, *SLC5A7*, a gene encoding the choline uptake transporter, was upregulated with creatine supplementation [*F*(1, 16) = 4.636, *P*_*TREAT*_ = 0.0469] (Figure 1D and Supplementary Table 2). Lastly, in the striatum, creatine treatment upregulated anti-apoptotic *BCL2* [*F*(1, 20) = 5.66, *P*_*TREAT*_ = 0.028], pro-inflammatory cytokine *IL-1*β [*F*(1, 20) = 5.80, *P*_*TREAT*_ = 0.026], *MPO*, a gene encoding myeloperoxidase protein, an inflammatory enzyme that triggers oxidative stress and neuroinflammatory processes [*F*(1, 17) = 12.34, *P*_*TREAT*_ = 0.003], and creatine kinase *CKMT* [*F*(1, 20) = 6.77, *P*_*TREAT*_ = 0.017] (Figure 1D and Supplementary Table 2).

#### 3.1.3. The combined effect of UCO on and creatine regional gene expression

Within the striatum, there was a UCO and creatine interaction effect on both *PPAR*γ [*F*(1, 20) = 5.01, *P*_*INT*_ = 0.037] and *PTGS1* [*F*(1, 20) = 4.41, *P*_*INT*_ = 0.049] with *post-hoc* test revealing that in the absence of UCO, both *PPAR*γ (1.7-fold; *P* = 0.042) and *PTGS1* (2.2-fold; *P* = 0.043) gene expression was increased in CrCon fetuses compared to SalCon fetuses (Figures 1B, D and Supplementary Table 2). Also in the striatum, there was a UCO and creatine interaction effect on the expression of pro-apoptotic *BAK1* [*F*(1, 20) = 9.12, *P*_*INT*_ = 0.0068], but *post-hoc* tests revealed no significant differences between groups (Supplementary Table 2).

In the hippocampus, there was a UCO and creatine interaction effect on *NOX1* [*F*(1, 15) = 7.33, *P*_*INT*_ = 0.016] expression, where in the absence of UCO, creatine treatment (CrCon) downregulated the expression of *NOX1* (compared to SalCon; *P* = 0.017; Supplementary Table 2). *OCLN* expression in the hippocampus was also affected by UCO and creatine treatment combined [*F*(1, 19) = 5.32, *P*_*INT*_ = 0.033], however, *post-hoc* tests revealed no significant differences between groups (Supplementary Table 2). For all the genes examined, no significant effect of creatine following UCO was revealed (Figure 1C).

### 3.2. Effects of global hypoxia and creatine supplementation on histopathology

#### 3.2.1. Cell death

**FIGURE 2:**
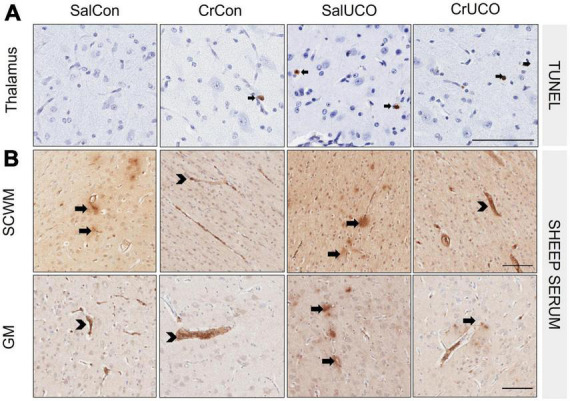
**(A)** Representative images of TUNEL-positive cells (black arrows) indicating cell death in thalamus. **(B)** Representative images of sheep serum extravasation from blood vessels into the brain parenchyma (arrows), and sheep serum contained within the blood vessels (arrowhead) in the subcortical white matter (SCWM) and cortical grey matter (GM). Scale bar represents 100 μm.

In addition to the increase in the density of TUNEL-positive cells within the hippocampal CA1-3 region reported previously in the same fetuses (Muccini et al., 2022), this study found that UCO increased cell death in the thalamus 72 h after hypoxia [*F*(1, 24) = 5.68; *P*_*UCO*_ = 0.025; Figure 2A and Table 2], compared to controls. No effect of UCO was observed in the caudate, CC or putamen. Neither creatine treatment alone nor creatine and UCO affected the density of TUNEL-positive cells for any brain region (Table 2).

**TABLE 2:** Neuropathological effects of umbilical cord occlusion (UCO) and/or creatine treatment.

Staining	Brain region	Group				2-Way ANOVA		
		**SalCon**	**CrCon**	**SalUCO**	**CrUCO**	***P*_UCO_**	***P*_TREAT_**	***P*_INT_**
TUNEL-positive cell density (cells/mm^2^)	Thalamus	1.35 ± 0.42	1.78 ± 0.90	2.21 ± 0.70	2.80 ± 1.68	0.025*	0.205	0.843
	Caudate	1.20 ± 0.52	1.27 ± 0.82	0.93 ± 0.50	1.14 ± 0.36	0.365	0.513	0.756
	CC	0.29 ± 0.72	0.36 ± 0.41	0.32 ± 0.37	0.40 ± 0.71	0.867	0.751	0.983
	Putamen	1.20 ± 0.72	1.23 ± 1.28	1.70 ± 1.14	1.63 ± 1.38	0.331	0.966	0.916
Sheep serum-positive blood vessels (average number)	SCWM	2.00 ± 1.23	0.64 ± 0.69	1.44 ± 0.98	0.79 ± 1.15	0.594	0.016*	0.594
	GM	0.71 ± 1.08	0.71 ± 1.08	1.75 ± 1.81	0.79 ± 1.08	0.682	0.047*	0.600
	PVWM	1.50 ± 0.84	0.07 ± 0.19	0.94 ± 1.35	0.71 ± 1.47	0.925	0.061	0.164
	Hipp	0.00 ± 0.00	0.00 ± 0.00	0.25 ± 0.71	1.14 ± 1.86	0.081	0.255	0.255
	Thalamus	0.33 ± 0.52	0.00 ± 0.00	0.53 ± 0.99	0.29 ± 0.76	0.368	0.283	0.869
	Caudate	0.33 ± 0.82	0.71 ± 0.81	0.63 ± 1.19	0.29 ± 0.76	0.847	0.953	0.316
	CC	0.17 ± 0.41	0.00 ± 0.00	0.13 ± 0.35	0.00 ± 0.00	0.845	0.181	0.845
	Internal capsule	0.00 ± 0.00	0.00 ± 0.00	0.38 ± 1.06	0.00 ± 0.00	0.420	0.420	0.420
	Putamen	0.00 ± 0.00	0.00 ± 0.00	0.00 ± 0.00	0.00 ± 0.00	–	–	–
NeuN-positive cell density (cells/mm^2^)	GM	671.67 ± 8.04	686.29 ± 58.31	667.50 ± 24.29	682.00 ± 32.42	0.911	0.695	0.997
	Hipp DG	3711.00 ± 768.95	3655.50 ± 1265.22	3708.38 ± 913.96	3555.50 ± 834.36	0.894	0.786	0.899
	Hipp CA1-3	697.00 ± 73.15	775.17 ± 161.27	716.63 ± 81.91	707.29 ± 95.27	0.821	0.747	0.682
	Thalamus	322.17 ± 64.67	354.83 ± 51.77	365.63 ± 55.21	293.43 ± 28.54	0.864	0.706	0.322
	Caudate	519.33 ± 41.01	602.83 ± 27.06	536.50 ± 32.31	565.00 ± 25.50	0.215	0.504	0.233
	Putamen	479.17 ± 62.11	501.67 ± 41.29	477.13 ± 22.90	398.67 ± 37.52	0.215	0.504	0.233
IBA-1-positive cell density (cells/mm^2^)	SCWM	111.04 ± 28.37	121.61 ± 27.75	162.03 ± 72.69	151.96 ± 61.13	0.056	0.990	0.615
	GM	50.83 ± 35.32	63.39 ± 25.84	61.25 ± 28.49	78.57 ± 43.03	0.326	0.253	0.853
	PVWM	148.96 ± 56.60	157.32 ± 25.85	172.50 ± 80.10	171.07 ± 63.12	0.428	0.882	0.834
	Hipp DG	84.37 ± 16.66	80.94 ± 46.16	117.71 ± 38.24	109.53 ± 49.68	0.055	0.709	0.878
	Hipp CA1-3	105.56 ± 69.66	138.10 ± 44.84	181.25 ± 174.67	247.62 ± 178.06	0.140	0.143	0.640
	Thalamus	54.86 ± 42.77	39.29 ± 39.74	60.94 ± 59.72	43.45 ± 23.06	0.763	0.334	0.955
	Caudate	110.42 ± 30.59	114.29 ± 59.07	104.17 ± 25.10	119.64 ± 43.48	0.978	0.546	0.716
	CC	83.33 ± 111.56	47.62 ± 0.48.52	62.50 ± 91.18	40.48 ± 41.79	0.640	0.338	0.819
	Internal capsule	132.64 ± 66.27	117.26 ± 40.50	129.17 ± 55.10	127.98 ± 43.68	0.856	0.678	0.722
	Putamen	154.17 ± 52.37	148.21 ± 45.89	126.04 ± 55.89	147.62 ± 68.29	0.509	0.718	0.526
IBA-1-positive area coverage (%)	SCWM	2.31 ± 1.79	2.53 ± 1.51	3.73 ± 2.50	3.85 ± 2.15	0.092	0.834	0.950
	GM	0.79 ± 1.39	1.34 ± 1.32	1.45 ± 1.70	2.09 ± 1.71	0.247	0.325	0.939
	PVWM	3.72 ± 1.38	4.17 ± 1.44	3.55 ± 1.40	5.01 ± 2.26	0.606	0.142	0.432
	Hipp DG	1.15 ± 0.61	1.57 ± 1.23	1.52 ± 1.35	1.88 ± 1.07	0.438	0.370	0.946
	Hipp CA1-3	1.04 ± 0.67	2.02 ± 1.82	2.48 ± 3.56	4.28 ± 3.28	0.085	0.190	0.694
	Thalamus	0.99 ± 0.95	1.11 ± 1.75	1.42 ± 2.20	1.79 ± 2.68	0.481	0.760	0.875
	Caudate	1.34 ± 0.60	1.00 ± 0.37	1.55 ± 0.69	2.32 ± 2.21	0.111	0.640	0.244
	CC	0.66 ± 0.80	0.45 ± 0.32	0.93 ± 1.71	1.01 ± 1.81	0.424	0.897	0.780
	Internal capsule	5.87 ± 6.80	3.71 ± 1.26	3.94 ± 2.83	6.75 ± 3.64	0.710	0.827	0.107
	Putamen	5.64 ± 5.55	5.46 ± 3.00	4.77 ± 4.06	6.26 ± 2.95	0.983	0.668	0.583
GFAP-positive cell density (cells/mm^2^)	SCWM	352.50 ± 50.62	400.00 ± 71.05	356.7 ± 115.84	329.29 ± 44.61	0.278	0.740	0.223
	GM	367.50 ± 40.25	344.11 ± 78.33	374.38 ± 75.52	426.79 ± 77.94	0.110	0.596	0.173
	PVWM	230.00 ± 61.04	230.36 ± 660.19	227.03 ± 86.36	217.50 ± 75.17	0.781	0.872	0.862
	Hipp DG	393.06 ± 143.36	385.12 ± 144.32	404.17 ± 160.77	589.29 ± 145.06	0.070	0.131	0.102
	Hipp CA1-3	544.47 ± 137.30	497.63 ± 113.21	589.58 ± 161.83	721.41 ± 238.96	0.049*	0.518	0.180
	Thalamus	387.50 ± 155.93	320.24 ± 123.10	368.75 ± 101.60	420.83 ± 113.45	0.389	0.872	0.213
	Caudate	411.11 ± 112.75	376.19 ± 82.35	385.94 ± 81.86	385.12 ± 77.24	0.811	0.599	0.616
	CC	197.22 ± 105.10	247.62 ± 71.64	266.67 ± 77.67	252.38 ± 40.17	0.210	0.537	0.273
	Internal capsule	252.78 ± 62.45	297.02 ± 23.90	277.09 ± 110.82	256.55 ± 98.66	0.801	0.712	0.317
	Putamen	316.67 ± 91.37	283.93 ± 57.10	300.52 ± 87.56	309.52 ± 69.63	0.874	0.690	0.485
GFAP-positive area coverage (%)	SCWM	31.17 ± 5.27	30.72 ± 7.30	31.41 ± 5.32	23.41 ± 8.91	0.188	0.119	0.161
	GM	17.48 ± 0.79	21.50 ± 8.97	18.62 ± 4.17	21.50 ± 8.97	0.220	0.915	0.085
	PVWM	29.76 ± 4.48	31.51 ± 6.63	29.72 ± 30.02	27.71 ± 7.93	0.391	0.953	0.401
	Hipp DG	22.65 ± 4.63	20.41 ± 4.81	22.01 ± 7.49	24.49 ± 10.50	0.545	0.966	0.408
	Hipp CA1-3	10.24 ± 3.18	10.57 ± 3.38	11.71 ± 4.96	16.82 ± 11.97	0.156	0.311	0.373
	Thalamus	16.08 ± 7.21	15.38 ± 4.61	20.22 ± 6.78	23.09 ± 9.27	0.039*	0.691	0.517
	Caudate	28.22 ± 7.13	28.01 ± 6.53	32.47 ± 6.92	27.25 ± 0.807	0.529	0.329	0.367
	CC	**18.84 ± 2.81**	26.38 ± 5.99	**28.35 ± 5.19^#^**	22.50 ± 7.59	0.208	0.700	0.005*
	Internal capsule	22.75 ± 4.91	19.83 ± 3.90	22.10 ± 4.50	19.86 ± 8.81	0.890	0.256	0.881
	Putamen	15.98 ± 4.66	14.50 ± 4.98	15.03 ± 3.96	14.83 ± 6.22	0.850	0.680	0.758
Olig-2-positive cell density (cells/mm^2^)	SCWM	1100 ± 298.04	1263.57 ± 341.59	1090.31 ± 336.68	1152.68 ± 346.06	0.635	0.382	0.695
	GM	269.79 ± 65.55	256.55 ± 66.97	279.95 ± 62.45	278.87 ± 77.45	0.537	0.785	0.817
	PVWM	184.04 ± 370.42	2026.07 ± 303.97	1900.78 ± 369.18	1717.68 ± 354.19	0.360	0.994	0.180
	Hipp DG	402.08 ± 164.97	338.69 ± 210.40	435.42 ± 190.71	368.45 ± 211.11	0.676	0.391	0.981
	Hipp CA1-3	319.45 ± 148.11	373.81 ± 232.51	364.58 ± 221.01	240.48 ± 190.73	0.574	0.656	0.260
	CC	1194.45 ± 181.56	1130.95 ± 296.18	1202.09 ± 337.53	1138.10 ± 349.56	0.950	0.587	0.998
	External capsule	1225.00 ± 343.63	1371.43 ± 463.65	1143.75 ± 395.51	1209.52 ± 408.57	0.440	0.499	0.797
CNPase-positive area coverage (%)	SCWM	47.50 ± 8.24	48.25 ± 8.67	47.47 ± 10.07	45.84 ± 14.70	0.769	0.915	0.774
	PVWM	59.22 ± 6.35	50.31 ± 8.87	55.36 ± 10.03	48.31 ± 12.25	0.435	0.041*	0.804
	Hipp DG	10.91 ± 0.599	9.32 ± 4.57	9.86 ± 7.73	12.42 ± 4.03	0.658	0.833	0.373
	Hipp CA1-3	10.98 ± 2.65	11.59 ± 6.22	10.75 ± 5.02	15.42 ± 5.85	0.407	0.226	0.350
	CC	47.62 ± 31.64	40.13 ± 26.78	45.05 ± 26.92	40.38 ± 13.91	0.907	0.543	0.888
	External capsule	58.89 ± 8.80	69.75 ± 11.14	57.00 ± 15.94	66.14 ± 9.31	0.571	0.048*	0.859
MBP-positive cell density (cells/mm^2^)	SCWM	491.88 ± 103.23	477.50 ± 111.92	447.50 ± 158.89	478.93 ± 119.87	0.662	0.862	0.641
	GM	87.85 ± 14.93	86.31 ± 19.65	94.79 ± 30.09	105.06 ± 17.30	0.137	0.606	0.486
	PVWM	583.33 ± 183.79	522.86 ± 197.82	468.28 ± 256.39	618.21 ± 140.41	0.895	0.552	0.169
	Hipp DG	146.53 ± 62.88	144.63 ± 71.63	152.08 ± 69.61	168.45 ± 71.11	0.582	0.785	0.731
	Hipp CA1-3	194.45 ± 70.45	226.19 ± 87.06	189.58 ± 93.41	216.67 ± 72.65	0.820	0.357	0.941
	CC	294.45 ± 113.37	361.90 ± 181.74	335.42 ± 165.10	423.81 ± 206.35	0.439	0.244	0.874
	External capsule	425.00 ± 143.27	454.76 ± 233.28	485.42 ± 164.61	530.95 ± 266.22	0.397	0.639	0.922
MBP-positive area coverage (%)	SCWM rostral	**20.05 ± 5.66**	**37.11 ± 10.76^&^**	27.87 ± 11.07	29.09 ± 10.99	0.979	0.025*	0.050*
	SCWM caudal	41.72 ± 9.37	39.82 ± 9.57	48.87 ± 20.70	46.71 ± 17.20	0.245	0.733	0.984
	GM	9.28 ± 1.82	10.65 ± 4.23	11.09 ± 1.71	10.27 ± 2.68	0.506	0.799	0.312
	PVWM	28.76 ± 6.64	39.62 ± 9.79	35.13 ± 12.86	35.84 ± 10.00	0.744	0.153	0.208
	Hipp DG	15.11 ± 8.42	11.61 ± 3.99	12.92 ± 7.19	13.96 ± 7.50	0.977	0.645	0.397
	Hipp CA1-3	20.14 ± 9.68	15.52 ± 8.68	16.69 ± 10.50	17.23 ± 8.48	0.820	0.582	0.469
	CC	17.07 ± 12.22	27.29 ± 13.79	26.98 ± 12.27	32.94 ± 26.73	0.249	0.232	0.749
	External capsule	19.28 ± 9.57	27.94 ± 8.29	27.76 ± 8.92	26.32 ± 14.21	0.398	0.374	0.217

Cell density (cells/mm^2^) and area coverage (%) analyses of immuno-positive staining assessed in saline control (SalCon; *n* = 6), creatine control (CrCon; n = 7), saline UCO (SalUCO; *n* = 8) and creatine UCO (CrUCO; *n* = 7). All data are expressed as mean ± SD and analysed by two-way ANOVA and Tukey’s multiple comparisons test. Significant main effects and interaction set at *P < 0.05. Significant differences between SalCon vs. CrCon indicated as ^&^*P* < 0.05. Significant differences between SalCon vs. SalUCO indicated as ^#^*P* < 0.05. SCWM, subcortical white matter; GM, grey matter; PVWM, periventricular white matter; Hipp DG, hippocampal dentate gyrus; Hipp CA1-3, hippocampal cornu ammonis 1-3; CC, corpus collosum. “-” indicated invalid data. Bold indicates significant main or interaction effects and groups in which a significant difference was found.

#### 3.2.2. Blood-brain barrier permeability

There were no effects of UCO with or without creatine on sheep serum extravasation within any brain region (Table 2). Within the SCWM and GM, there was a decrease in the average number of sheep serum-positive blood vessel profiles with creatine treatment alone [*F*(1, 24) = 6.71, *P*_*TREAT*_ = 0.016 and *F*(1, 24) = 4.39, *P*_*TREAT*_ = 0.047, respectively, Figure 2B and Table 2].

#### 3.2.3. Mature neurons

**FIGURE 3:**
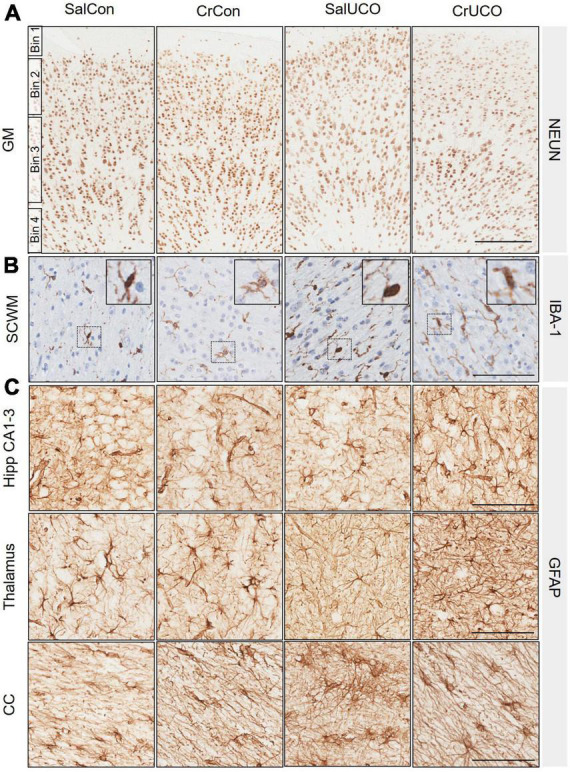
**(A)** Representative images of NeuN-positive cells indicating mature neurons in the cortical grey matter (GM). GM counts were conducted in four bins roughly equivalent to cortical layer I (Bin 1), layer II and III (Bin 2), layer IV and V (Bin 3), and layer VI (Bin 4). No significant differences of bins were found between groups, therefore data were combined and presented as a total average for GM. **(B)** Representative images of IBA-1-positive cells indicating microglia in the subcortical white matter (SCWM). Insert demonstrates ramified microglia. **(C)** Representative images of glial fibrillary acidic protein (GFAP)-positive cells and processes indicating astrocytes in the dorsal hippocampal (Hipp) CA1-3, thalamus and corpus callosum (CC). Scale bar represents 100 μm.

No group differences were found within each GM binned analysis and therefore NeuN cell density is expressed as a total summation within the GM. The cell density of NeuN-positive neurons was similar in all groups for all brain regions examined (Figure 3A and Table 2).

**FIGURE 4:**
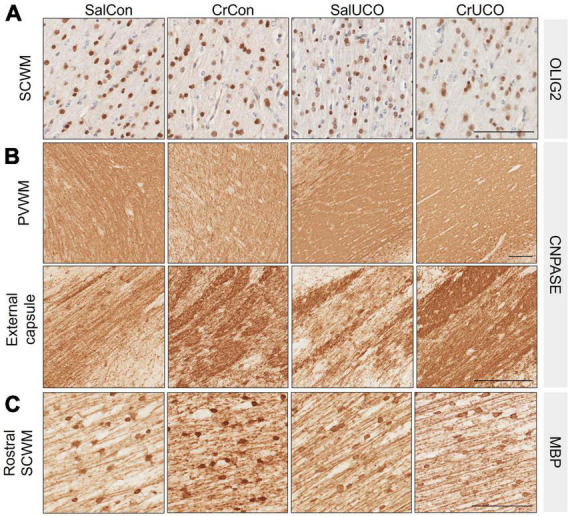
**(A)** Representative images of Olig2-positive oligodendrocytes in the subcortical white matter (SCWM). **(B)** Representative images of CNPase-positive staining in the periventricular white matter (PVWM) and external capsule. **(C)** Representative images of MBP-positive oligodendrocytes and fibers indicating mature myelination in the rostral SCWM. Scale bar represents 100 μm.

#### 3.2.4. Neuroinflammation: Microglia and astrocytes

There were no significant changes in the cell density and area coverage of IBA-1-positive staining within any of the brain regions (Table 2). There was, however, a near to significant trend for a UCO effect on increasing IBA-1 cell density in both the SCWM and hippocampal DG (*P*_*UCO*_ = 0.056 and *P*_*UCO*_ = 0.055, respectively, Figure 3B and Table 2).

Umbilical cord occlusion increased GFAP-positive cell density in the hippocampal CA1-3 region [*F*(1, 24) = 4.31, *P*_*UCO*_ = 0.049; Figure 3C and Table 2] with a trend to also increase astrocyte density within the hippocampal DG [*F*(1, 24) = 3.60; *P*_*UCO*_ = 0.070; Table 2]. UCO also increased GFAP-positive area coverage in the thalamus compared to controls [*F*(1, 24) = 4.77, *P*_*UCO*_ = 0.039; Table 2 and Figure 3C]. In the CC of saline-treated fetuses, UCO increased GFAP-positive area coverage 50.5% compared to SalCon (*P* = 0.03). This increase in GFAP-positive area coverage caused by the UCO was reduced in creatine-treated fetuses by 20.6% [*F*(1, 24) = 9.468,*P*_*INT*_ = 0.005; Table 2 and Figure 3C].

#### 3.2.5. Oligodendrocytes and myelination

White matter development and integrity was assessed using oligodendrocyte lineage and myelination markers (see Figure 4). There was no effect of UCO or creatine treatment on Olig-2-positive cell density in any brain region (Table 2 and Figure 4A). Analysis of the CNPase-positive area coverage revealed that creatine treatment decreased CNPase-positive area coverage in the PVWM by 14.5% [*F*(1, 24) = 4.65, *P*_*TREAT*_ = 0.041], but in contrast, the CNPase area coverage was increased by 18% in the external capsule [*F*(1, 22) = 4.37, *P*_*TREAT*_ = 0.048; Table 2 and Figure 4B]. Assessment of mature oligodendrocytes using MBP-positive cell density revealed no effects of creatine nor UCO (Table 2). However, MBP-positive area coverage in the SCWM showed a rostro-caudal difference, with a significant creatine treatment main effect as well as a significant interaction of treatment and UCO at the more rostral levels of the brain [*F*(1, 24) = 5.70, *P*_*TREAT*_ = 0.025; F(1, 24) = 4.28, *P*_*INT*_ = 0.050; Table 2 and Figure 4C]. *Post-hoc* test revealed that creatine treatment alone significantly increased the MBP-positive area coverage in the SCWM (*P* = 0.027).

## 4. Discussion

This study examined brain region-specific neuropathology 72 h after a brief (10 min) *in utero* acute hypoxia at a gene expression and histopathology level, and assessed whether creatine treatment commenced before the hypoxic episode affected these outcomes. The cortical GM, thalamus and hippocampus were particularly susceptible to neuropathology following UCO irrespective of creatine treatment, with increased evidence of cell death and neuroinflammation, and changes in expression of many genes associated with the regulation of injury responses, the vasculature, and mitochondrial function. Creatine treatment prior to hypoxia did not have a large impact on any of the indices of neuropathology induced by the UCO, except for reducing the astrogliosis in the corpus callosum. Interestingly, there were numerous effects of creatine treatment alone on gene expression, particularly of apoptotic, inflammatory, and oxidative stress-related pathways in the cortical GM, hippocampus and striatum. However, these transcriptional changes were not reflected at a histopathological level.

### 4.1. Regional vulnerability to UCO and potential consequences

It was evident that specific brain regions, namely, the cortical GM, thalamus, and hippocampus were particularly vulnerable to the mild hypoxia caused by UCO in these near-term fetal sheep. This is consistent with clinical presentations in which term human infants have a predilection for brain injury patterns involving watershed regions; i.e., cortical gray matter and the basal ganglia (Miller et al., 2005). In experimental newborn animals, the hippocampus also appears to be particularly vulnerable to hypoxic-ischemic injury (Martin et al., 1997; Roland et al., 1998; Kreisman et al., 2000). This pattern of regional vulnerability in the term newborn is thought to be due to the presence of dense neuronal populations, their high energy requirements and inherent susceptibility to excitotoxicity, oxidative stress, and apoptosis (Billiards et al., 2006).

Whilst the hypoxic injury evident at 72 h post-UCO was relatively mild there were indications of persisting injury within the GM and thalamus. Modulation of neuropathological processes such as apoptosis, inflammation, perturbed energy metabolism and angiogenesis following hypoxia are predominately mediated by HIF-1α induction (Liang et al., 2018). In the present study, there were no significant changes in *HIF-1*α gene expression 72 h after UCO. Despite this, after UCO we observed reduced gene expression in the cortical GM and thalamus of factors downstream to HIF-1α, such as the pluripotent transcription factor *NF*κ*B1*, pleotropic cytokine *TGF-*β, synaptogenic protein *NRG1*, the pleotropic ligand-inducible nuclear transcription factor *PPAR*γ, and vascular growth factors V*EGFA* and *ANGPT2* – all key factors associated with HIF-1α function (Klempt et al., 1992; Zhu et al., 2002; Pichiule et al., 2004; Zhao et al., 2009; Akwii et al., 2019; Navarro-González et al., 2019; Yoo et al., 2019). Given their individual roles in maintaining cellular homeostasis and neuroprotection, the decreased expression of these genes may suggest reduced cellular protection and the potential for persisting cellular perturbations. It is important to note that the physiological changes provoked by UCO had all returned to pre-UCO levels by 24 h post-UCO (Tran et al., 2021b,^2022^), so that decreased gene expression after 72 h suggests persisting cellular effects within the cortical GM and thalamus following acute *in utero* hypoxia, and perhaps an increased susceptibility to the effects of any subsequent hypoxic episode that might arise before or after birth.

We observed minimal histopathology and no effect on gene expression following UCO in WM regions, contrary to our previous indications of disturbed mitochondrial respiration in the WM of the same fetuses (Muccini et al., 2022). We were unable to separate cellular populations within our procedure for mitochondria isolation in this previous study (Muccini et al., 2022), and therefore cannot be certain which cell type may, or may not have had, disruption of mitochondrial respiration. Though, it has been shown that mature oligodendrocytes are not particularly vulnerable to perturbations to the electron transport chain (Fünfschilling et al., 2012). Moreover, given the trend for increased microglial density within the SCWM, it is likely another cell type within the WM were affected by the UCO at 72 h which requires further investigation. Collectively, the neuropathology present 72 h after even a mild hypoxic insult suggests a potential progression or development of delayed neurodegeneration over a long period of time (Miller and Ferriero, 2009; Baburamani et al., 2021).

### 4.2. Creatine prophylaxis protection

We observed that creatine treatment reduced astrocyte area coverage following UCO in the corpus callosum, suggesting a potential protective effect following hypoxic injury (Raivich et al., 1999; Chiareli et al., 2021). The reason why this creatine effect was only seen in the corpus callosum, and not elsewhere such as in the thalamus and hippocampus and white matter regions, requires further elucidation. The finding is of clinical interest as callosal injuries have been identified in newborns diagnosed with HIE, and this kind of injury contributes to poor neurological outcomes (Mañeru et al., 2003; Epelman et al., 2012). It is possible that this protective effect of creatine in the corpus callosum may be present only after mild and brief hypoxia as induced in this study, and therefore further study of a more severe hypoxic injury is warranted.

### 4.3. Potential influence of creatine prophylaxis on brain development and function

In this study, the continuous infusion of creatine to the fetus for 13 days resulted in multiple effects that indicate changes to the innate immune system. We found that creatine treatment downregulated gene expression of *TLR3* in the GM. A similar downregulation of toll-like receptor expression has been observed *in vitro* in a mouse macrophage cell line (Leland et al., 2011). Moreover, in the GM and striatum we observed upregulated expression of major cytokine genes (*TNF*α, *IL-6* and *IL-1*β), of myeloperoxidase (*MPO)*, a critical inflammatory enzyme involved in triggering oxidative stress responses, and of *PTGS1*, the gene encoding cyclooxygenase 1 involved in producing prostaglandins. The increased expression of pro-inflammatory and oxidative stress mediators suggests an increasing pro-inflammatory environment as has been indicated by studies in the lungs showing that creatine supplementation can exacerbate pulmonary inflammation by inducing airway inflammation, remodeling and hyper-responsiveness (Vieira et al., 2007; Garcia et al., 2019).

However, alternatively, these results could actually indicate changes in the physiological resting state that could be protective, as observed in studies where creatine supplementation has anti-inflammatory effects following *in vitro* oxidative and *in vivo* exercise injury (Nomura et al., 2003; Bassit et al., 2008). Creatine may potentially aid in controlling particular proinflammatory states (Bredahl et al., 2021), which may have implications for perinatal diseases such as cerebral palsy (Gouveia et al., 2022). The downregulation of NADPH oxidase 1 (*NOX1)* expression in the hippocampus supports this hypothesis of improved resistance to hypoxic injury, as genetic deletion or inhibition of NOX1 reduces ischemic lesions, preserves BBB integrity (Kahles et al., 2010), reduces ROS production (Coyoy et al., 2008), and reduces the extent of neuronal degeneration after stroke (Choi et al., 2015). Regardless, whether these gene expression changes are reflected in changes in the expressed proteins needs to be ascertained. Importantly, we did not find protein immunostaining evidence that creatine supplementation alone increases the presence of neuroglia (microglia and astrocytes) nor increases markers of oxidative stress, as reported previously (Tran et al., 2022). Therefore, these creatine effects on inflammatory pathways need to be investigated further, as we cannot conclusively say if these changes have adverse or protective effects overall. Though, the latter is more likely as these creatine-induced inflammatory transcriptional changes did not present as adverse changes in protein expression (i.e., no changes in microglia and astrocyte cell density and improved vascular integrity).

Importantly, we show here for the first time that creatine supplementation irrespective of hypoxia did have effects on creatine metabolism in the fetal brain. Creatine supplementation significantly increased gene expression for mitochondrial creatine kinase (CKMT) in the striatum, with a trend for an increase in the GM and hippocampus but decreased expression in the WM as reported previously (Muccini et al., 2022). These results complement the increases in total creatine content found within the striatum, GM, and hippocampus, and no increase in the WM in the same fetuses (Tran et al., 2021b). The increased expression of *CKMT* is consistent with an increased need for creatine phosphorylation to maintain the 1:3 ratio of creatine to phosphocreatine within cells as creatine levels increase (Wallimann et al., 1992). Indeed, endogenous creatine concentrations are known to be higher in GM regions (McLean et al., 2000), and CKMT is more highly expressed in neuronal populations due to their high oxidative metabolism (Tachikawa et al., 2004; Lowe et al., 2013). Moreover, the decreased expression of *CKMT* but no change in creatine tissue content in the WM is consistent with the findings of Chamberlain et al. (Chamberlain et al., 2017) who reported that creatine treatment of primary oligodendrocyte cultures enhanced mitochondria density and ATP production, suggesting a reduced need for creatine phosphorylation and therefore a decreased need for *CKMT* expression. Moreover, in the present study there were no expression changes to *SLC6A8*, the gene that encodes the creatine transporter with creatine supplementation, consistent with other reports (Brault et al., 2003; Tarnopolsky et al., 2003; Andre et al., 2015).

Interestingly, there were also indications that prolonged creatine supplementation may have long term effects on cellular function. In the hippocampus creatine supplementation increased *SLC5A7* expression. *SLC5A7* encodes the high-affinity choline transporter that maintains cholinergic neurotransmission by supporting the synthesis of acetylcholine (Okuda et al., 2000). Cholinergic input to the hippocampus is involved in cognitive processing and extinction memory (Ballinger et al., 2016; Knox and Keller, 2016). Recently, multiple studies have investigated the use of creatine supplementation to enhance cognitive performance which, in part, may be due to increased choline availability and utilization see review (Dolan et al., 2019). We also observed reduced CNPase area coverage in the PVWM, increased MBP-positive area coverage in the rostral SCWM, and increased CNPase in the external capsule after the 13 days of creatine treatment irrespective of hypoxia. Creatine kinase activity and density is inherently high in oligodendrocytes suggesting their cellular function and response to injury could also be modified by increased creatine levels (Manos and Bryan, 1993). Appropriate myelinogenesis is integral for the establishment and consolidation of functional neuronal networks (Mathis et al., 2001; Doretto et al., 2011), and our study indicates a potential for creatine to enhance myelination in the developing brain. The increased amount of mature myelin observed in creatine-treated fetuses was primarily within the rostral SCWM section, i.e., post-central sulci, involving the somatosensory cortex and not in the caudal SCWM section, which includes the occipital lobe cortex (see Supplementary Figure 1). This finding suggests an effect on sensory information processing. Early and accelerated abnormal maturation of white matter has been observed in adolescents with autism spectrum disorder (Ben Bashat et al., 2007), therefore the increased myelin maturation may indicate an increased susceptibility to neurodevelopmental disorders with sensory disabilities. However, recent work supports the idea that increased *in utero* myelin maturation is coupled with a reduced rate of maturation after birth, meaning overall myelination remains balanced (Grotheer et al., 2022), and thus the increased myelin maturation may have no long-term effects or rather improved cognition (Chevalier et al., 2015). Regardless, further studies with and without increased *in utero* creatine exposure are required to understand these myelin maturation processes and subsequent consequences on neurodevelopment.

Together, this study highlights potential neuroprotective but also potentially adverse neurodevelopmental effects of increased creatine exposure *in utero*. Human data demonstrates that there are no toxicity or adverse risks associated with high and prolonged exposure to oral creatine monohydrate in non-pregnant females (de Guingand et al., 2020), and investigations in spiny mice that followed offspring until sexual maturity (3 months of age) found no difference in development between pups exposed to creatine during pregnancy and controls (LaRosa et al., 2016; Ellery et al., 2017). However, continuing to include assessments of the potential effects of creatine supplementation alone in preclinical studies will provide important safety and efficacy data to ensure effective and appropriate translation of promising findings.

### 4.4. Limitations of this study

A limitation of this study is the assessment of neuropathology at a single time point post-UCO and creatine treatment. Indeed, the pathogenesis of hypoxia-ischemia encephalopathy involves an evolution of injury and recovery over time. Therefore, an assessment at a single time point cannot completely reflect the dynamic and complex development of injury and repair. By allowing 72 h to elapse after UCO there may already be partial or near complete restoration of cellular homeostasis, by which time some of the effects of creatine treatment would no longer be apparent. Indeed, a follow-up analysis of protein levels is needed to confirm the biological importance of observed gene expression changes. However, as differences in gene expression persisted 72 h post UCO and 13 days of creatine infusion suggests that there is likely changes to protein levels and thus cellular metabolism and function.

Also important to note is that this study evidently reflects a quite mild hypoxic insult given the absence of profound brain damage (Tran et al., 2021b). Whilst mild neonatal HIE cases are now increasingly recognized as a significant risk factor for an impaired neurodevelopmental trajectory (Conway et al., 2018), clinically, abnormalities are detectable by cerebral magnetic resonance imaging by 3 days of life (Walsh et al., 2017). Therefore, the 10 min UCO within this cohort of fetal sheep may not properly reflect these clinical mild HIE cases that are at risk of developing into cases of only mild neurodevelopmental delays. Nonetheless, the creatine effects irrespective of UCO found within this study may reflect neuroprotective effects that were not identifiable due to the mild mature of the hypoxic insult and the timing of assessment. Future studies assessing different time points (i.e., during primary, secondary, and tertiary energy failure) and following up assessments of protein levels reflecting the gene expression data are needed. Moreover, extending the analyses presented in this study into a more moderate-to-severe model of hypoxia would not only be clinically relevant but may also provide a better understanding and consolidation of the potential neuroprotective effects of creatine.

Another limitation of this study was the small sample size akin to large animal studies. Small group sizes may reduce the effect size of observed differences between groups and can underpower effects that would have otherwise been significant with larger group sizes. Nonetheless, this study has highlighted multiple cellular pathways affected by UCO and creatine, and in detail investigations are now required to elucidate these findings.

Finally, in this study it was necessary to infuse creatine directly into the fetus, as the ovine placenta does not transfer creatine in a maternal-fetal direction (Baharom et al., 2017). It is possible that, in human studies where it would be envisaged that fetal creatine supplementation would follow increased maternal intake of creatine, that there are effects on the placenta that may or may not improve fetal health. The full clinical evaluation of creatine supplementation in an obstetric population is yet to be undertaken.

## 5. Conclusion

In the present study, brain region-specific changes of mRNA expression and histopathology were present 72 h after a transient, mild global hypoxia in the near-term fetal sheep. Hypoxia resulted in significant astrogliosis and down-regulation of genes involved in cellular regulation, vasculature integrity and mitochondrial dynamics in the cortical GM and thalamus. Creatine supplementation ameliorated astrogliosis within the corpus callosum caused by hypoxia, however, no other obvious protective effects were found. Of particular interest is that creatine supplementation alone induced transcriptional changes in apoptotic and inflammatory pathways in the cortical gray matter, striatum and hippocampus, with evidence of increased myelination. This creatine effect warrants further investigation, as understanding any effects of creatine supplementation on normal fetal brain development will be essential if the proposal to give creatine prophylactically to pregnant women is to be a realistic clinical option.

## Data availability statement

The original contributions presented in this study are included in the article/Supplementary material, further inquiries can be directed to the corresponding author.

## Ethics statement

The animal study was reviewed and approved by Monash Medical Centre Animal Ethics Committee.

## Author contributions

NT, NH, AM, RS, DW, MT, and SE: study conception. NT, NH, MT, and SE: methodology. NT, AM, NH, and SE: data curation. NT, AM, and NH: data analysis. NT: writing—original draft preparation. NT, NH, RS, DW, MT, and SE: writing—review and editing. All authors contributed to the article and approved the submitted version.
