# Supplementary material for: Creatine in the fetal brain: A regional investigation of acute global hypoxia and creatine supplementation in a translational fetal sheep model
Source: Front Cell Neurosci. 2023 Mar 30;17:1154772. doi: 10.3389/fncel.2023.1154772 (PMC10097948; doi:10.3389/fncel.2023.1154772)
Supplement: Supplementary file 1 [file Data_Sheet_1.docx]

***Supplementary Material***

**Creatine in the fetal brain: a regional investigation of acute global hypoxia and creatine supplementation in a translational fetal sheep model**

Nhi T Tran*, Anna M Muccini, Nadia Hale, Mary Tolcos, Rod J Snow, David W Walker, Stacey J Ellery

*** Correspondence:** Nhi Thao Tran: nhi.tran@hudson.org.au

1. **Supplementary Figures and Tables**
   1. **Supplementary Figures**
      1. **Supplementary Figure 1**

Fetal sheep brain histology: Regions of interest. Immunohistochemistry was conducted on two sections of the right cerebral hemisphere of the fetal sheep brain with one taken posterior to the Ansate sulcus (i.e., Cx4) and one taken at the level of the thalamus and hippocampus (i.e., Cx7). Regions analysed include dorsal hippocampal CA1-3 (**CA1-3**): green; corpus collosum (**CC**): purple; dorsal hippocampal dentate gyrus (**DG**): fuchsia; external capsule (**EC**): black; cortical grey matter (**GM**): fluorescent green; internal capsule (**IC** (white matter striations only)); dark green; putamen (**Put**): orange; periventricular white matter (**PVWM**): red, subcortical white matter (**SCWM**): yellow; caudate (**Ca**): olive; thalamus (**Tha**): brown.


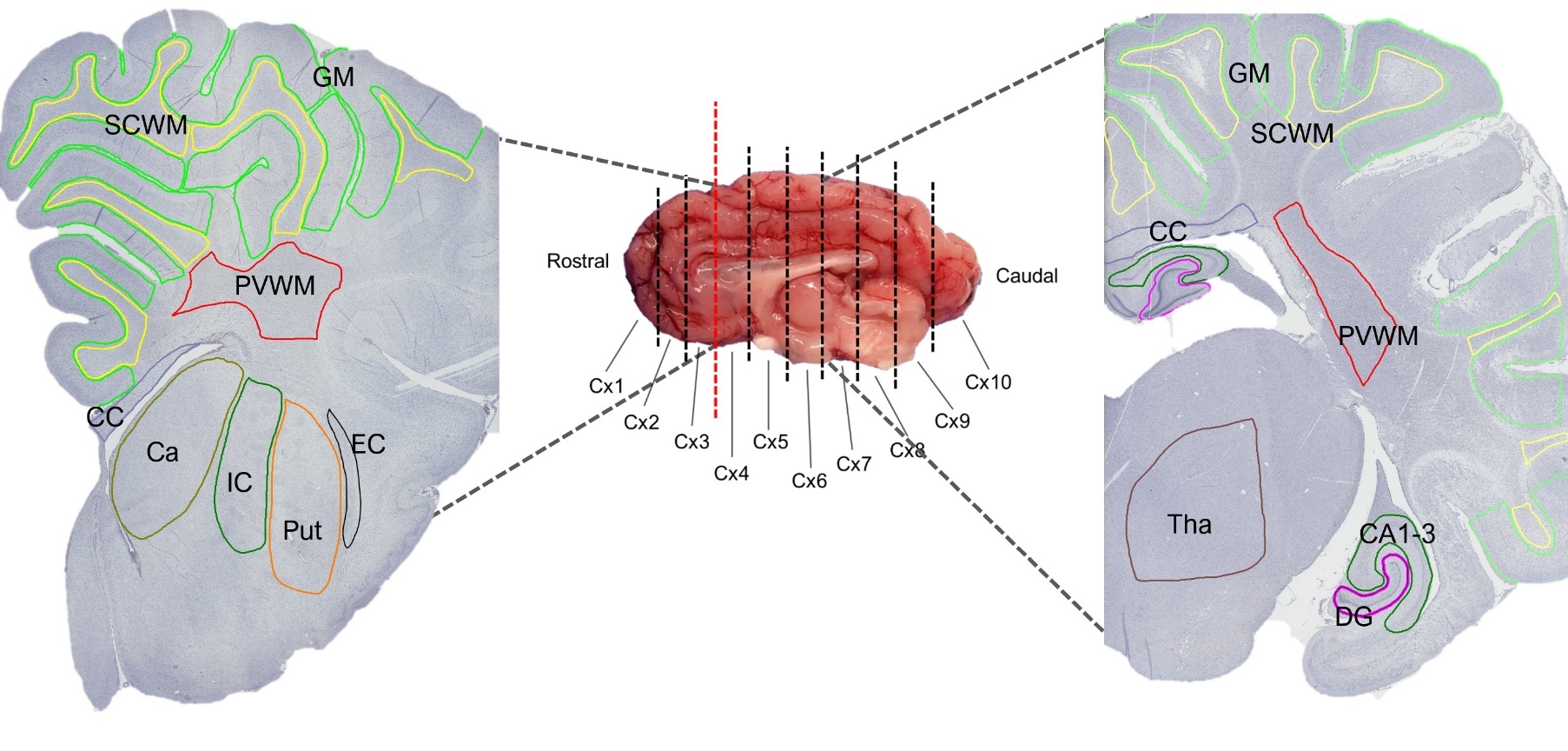


- 1. **Supplementary Tables**
     1. **Supplementary Table 1:**

Summary of finalized protocols for immunohistochemistry stains conducted. **BSA:** bovine serum albumin; **CNPase:** 2',3'-Cyclic-nucleotide 3'-phosphodiesterase; **GFAP:** Glial fibrillary acidic protein; **IBA-1:** Ionized calcium binding adaptor molecule-1; **MBP:** Myelin basic protein; **Olig-2:** Oligodendrocyte transcription factor 2; **PBS:** phosphate buffer.

| **Antibody** | **Detects** | **Antigen retrieval** | **Primary Antibody (1°Ab)** | **1°Ab diluent** | **[1°Ab]** | **Secondary Antibody (2°Ab)** | **2°Ab diluent** | **[2°Ab]** | **Counterstain** |
| --- | --- | --- | --- | --- | --- | --- | --- | --- | --- |
| Sheep serum | Serum protein within sheep blood | N/A | Rabbit anti-Sheep serum (Sigma Aldrich, USA) | 2% fish gelatin PBS | 1:700 | Goat biotinylated anti-rabbit IgG | PBS | 1:200 | N/A |
| NeuN | Mature neurons | Citrate Buffer (10 mM Tri-sodium citrate in dH2O, pH 6.0; Sigma Aldrich) | Mouse anti-NeuN (Millipore, Germany) | DAKO diluent (Dako, USA) | 1:500 | Goat biotinylated anti-mouse IgG (Vector Labs, USA) | PBS | 1:200 | N/A |
| IBA-1 | Microglia | Citrate Buffer | Rabbit anti-IBA-1 (Wako Chemicals, USA) | 1% BSA PBS | 1:1000 | Goat biotinylated anti-rabbit IgG (Vector Labs, USA) | 1% BSA in PBS | 1:200 | Hematoxylin |
| GFAP | Astrocytes | Proteinase K (Promega, USA) | Rabbit anti-GFAP (Dako, USA) | DAKO diluent | 1:500 | Goat biotinylated anti-rabbit IgG | PBS | 1:200 | N/A |
| Olig-2 | Oligodendrocytes | Citrate Buffer | Rabbit anti-Olig-2 (Millipore, Germany) | DAKO diluent | 1:500 | Goat biotinylated anti-rabbit IgG | PBS | 1:200 | Hematoxylin |
| CNPase | pre-myelinating and myelinating oligodendrocytes | Citrate Buffer | Mouse anti-CNPase (Sigma Aldrich, USA) | DAKO diluent | 1:500 | Goat biotinylated anti-mouse IgG | PBS | 1:200 | N/A |
| MBP | Mature myelin and oligodendrocytes | Citrate Buffer | Rabbit anti-MBP (Chemicon International, USA) | DAKO diluent | 1:200 | Goat biotinylated anti-rabbit IgG | PBS | 1:200 | N/A |

- - 1. **Supplementary Table 2:**

Summary of mRNA expression and statistical outputs. Data are average log10-transformed 2^−ΔΔCT^. Data are expressed relative to the geomean of housekeeping genes (*RPL32,* *RPS16* and *OAZ1*) and shown relative to saline control fetuses. Saline control (SalCon) n=3-5, creatine control (CrCon) n=4-7, saline UCO (SalUCO) n=6-8 and creatine UCO (CrUCO) n=5-6. Gene expression was measured within the cortical grey matter (GM), white matter (WM), hippocampus (Hipp), striatum, and thalamus. All data are expressed as mean ± SD and analysed by 2-way ANOVA and Tukey’s multiple comparisons test. Significant main effects and interaction set at **P*<0.05. Significant differences between SalCon vs. CrCon indicated as ^&^*P*<0.05.

| **Cell transcriptional factors** | | | | | | | | | | | | | | | | | | | | | | | | | | | | | | | | | | | | | | | | | |  |  |
| --- | --- | --- | --- | --- | --- | --- | --- | --- | --- | --- | --- | --- | --- | --- | --- | --- | --- | --- | --- | --- | --- | --- | --- | --- | --- | --- | --- | --- | --- | --- | --- | --- | --- | --- | --- | --- | --- | --- | --- | --- | --- | --- | --- |
| **Gene** | | | **Brain Region** | | | **Group** | | | | | | | | | | | | | | | | | | | | | | | | | | **2-Way ANOVA** | | | | | | | | | |  |  |
|  |  |  |  |  |  | **SalCon** | | | | | | **CrCon** | | | | | | | | | | | | **SalUCO** | | **CrUCO** | | | | | | ***P_UCO_*** | | | | ***P_TREAT_*** | | | | ***P_INT_*** | |  |  |
| *HIF1-α* | | | Striatum | | | 0.000 ± 0.223 | | | | | | 0.202 ± 0.144 | | | | | | | | | | | | 0.204 ± 0.208 | | 0.147 ± 0.145 | | | | | | 0.336 | | | | 0.351 | | | | 0.102 | |  |  |
|  | | | Thalamus | | | 0.000 ± 0.071 | | | | | | 0.224 ± 0.364 | | | | | | | | | | | | -0.024 ± 0.097 | | -0.013 ± 0.170 | | | | | | 0.197 | | | | 0.241 | | | | 0.290 | |  |  |
| *TGF-β* | | | GM | | | 0.000 ± 0.017 | | | | | | -0.076 ± 0.099 | | | | | | | | | | | | -0.151 ± 0.136 | | -0.081 ± 0.06 | | | | | | 0.081 | | | | 0.944 | | | | 0.103 | |  |  |
|  | | | WM | | | 0.000 ± 0.195 | | | | | | -0.183 ± 0.145 | | | | | | | | | | | | -0.044 ± 0.152 | | -0.155 ± 0.088 | | | | | | 0.913 | | | | 0.055 | | | | 0.622 | |  |  |
|  | | | Hipp | | | 0.000 ± 0.114 | | | | | | 0.094 ± 0.150 | | | | | | | | | | | | 0.022 ± 0.095 | | 0.051 ± 0.096 | | | | | | 0.836 | | | | 0.227 | | | | 0.512 | |  |  |
|  | | | Striatum | | | 0.000 ± 0.144 | | | | | | 0.109 ± 0.110 | | | | | | | | | | | | -0.005 ± 0.207 | | -0.016 ± 0.173 | | | | | | 0.350 | | | | 0.477 | | | | 0.385 | |  |  |
|  | | | Thalamus | | | 0.000 ± 0.095 | | | | | | 0.039 ± 0.123 | | | | | | | | | | | | -0.250 ± 0.213 | | -0.103 ± 0.161 | | | | | | **0.011*** | | | | 0.193 | | | | 0.445 | |  |  |
| *NFκB1* | | | GM | | | 0.000 ± 0.032 | | | | | | -0.086 ± 0.147 | | | | | | | | | | | | -0.275 ± 0.214 | | -0.119 ± 0.165 | | | | | | **0.044*** | | | | 0.628 | | | | 0.106 | |  |  |
|  | | | WM | | | 0.000 ± 0.120 | | | | | | -0.261 ± 0.312 | | | | | | | | | | | | -0.005 ± 0.211 | | -0.100 ± 0.104 | | | | | | 0.453 | | | | 0.097 | | | | 0.426 | |  |  |
|  | | | Hipp | | | 0.000 ± 0.100 | | | | | | -0.084 ± 0.063 | | | | | | | | | | | | -0.011 ± 0.097 | | -0.117 ± 0.334 | | | | | | 0.762 | | | | 0.205 | | | | 0.877 | |  |  |
|  | | | Striatum | | | 0.000 ± 0.299 | | | | | | 0.327 ± 0.120 | | | | | | | | | | | | 0.079 ± 0.463 | | 0.219 ± 0.173 | | | | | | 0.912 | | | | 0.079 | | | | 0.467 | |  |  |
|  | | | Thalamus | | | 0.00 ± 0.185 | | | | | | 0.007 ± 0.090 | | | | | | | | | | | | -0.456 ± 0.276 | | -0.251 ± 0.241 | | | | | | **0.002*** | | | | 0.295 | | | | 0.325 | |  |  |
| *NRG1* | | | GM | | | 0.000 ± 0.059 | | | | | | -0.049 ± 0.153 | | | | | | | | | | | | -0.066 ± 0.277 | | -0.02 ± 0.072 | | | | | | 0.818 | | | | 0.979 | | | | 0.555 | |  |  |
|  | | | WM | | | 0.000 ± 0.192 | | | | | | -0.154 ± 0.143 | | | | | | | | | | | | -0.002 ± 0.188 | | -0.108 ± 0.012 | | | | | | 0.772 | | | | 0.104 | | | | 0.755 | |  |  |
|  | | | Hipp | | | 0.000 ± 0.051 | | | | | | 0.052 ± 0.175 | | | | | | | | | | | | 0.121 ± 0.091 | | 0.112 ± 0.085 | | | | | | 0.072 | | | | 0.659 | | | | 0.531 | |  |  |
|  | | | Striatum | | | 0.000 ± 0.136 | | | | | | 0.117 ± 0.163 | | | | | | | | | | | | 0.130 ± 0.282 | | 0.084 ± 0.245 | | | | | | 0.596 | | | | 0.702 | | | | 0.376 | |  |  |
|  | | | Thalamus | | | 0.000 ± 0.229 | | | | | | 0.232 ± 0.353 | | | | | | | | | | | | -0.205 ± 0.203 | | -0.170 ± 0.331 | | | | | | **0.024*** | | | | 0.297 | | | | 0.436 | |  |  |
| *RCAN1* | | | GM | | | 0.000 ± 0.051 | | | | | | -0.029 ± 0.096 | | | | | | | | | | | | -0.035 ± 0.123 | | -0.019 ± 0.113 | | | | | | 0.789 | | | | 0.892 | | | | 0.640 | |  |  |
|  | | | WM | | | 0.000 ± 0.155 | | | | | | -0.074 ± 0.135 | | | | | | | | | | | | 0.016 ± 0.116 | | -0.046 ± 0.086 | | | | | | 0.708 | | | | 0.265 | | | | 0.918 | |  |  |
|  | | | Hipp | | | 0.000 ± 0.051 | | | | | | -0.009 ± 0.094 | | | | | | | | | | | | -0.030 ± 0.089 | | -0.009 ± 0.112 | | | | | | 0.698 | | | | 0.869 | | | | 0.694 | |  |  |
|  | | | Striatum | | | 0.000 ± 0.124 | | | | | | 0.196 ± 0.125 | | | | | | | | | | | | 0.177 ± 0.148 | | 0.192 ± 0.099 | | | | | | 0.119 | | | | 0.059 | | | | 0.103 | |  |  |
|  | | | Thalamus | | | 0.000 ± 0.185 | | | | | | -0.012 ± 0.113 | | | | | | | | | | | | -0.117 ± 0.108 | | -0.139 ± 0.173 | | | | | | 0.056 | | | | 0.773 | | | | 0.934 | |  |  |
| *COL1A1* | | | GM | | | 0.000 ± 0.073 | | | | | | -0.122 ± 0.131 | | | | | | | | | | | | 0.017 ± 0.258 | | -0.146 ± 0.114 | | | | | | 0.963 | | | | 0.079 | | | | 0.795 | |  |  |
|  | | | WM | | | 0.000 ± 0.145 | | | | | | -0.082 ± 0.208 | | | | | | | | | | | | 0.094 ± 0.206 | | -0.170 ± 0.185 | | | | | | 0.972 | | | | 0.066 | | | | 0.318 | |  |  |
|  | | | Hipp | | | 0.000 ± 0.211 | | | | | | -0.077 ± 0.357 | | | | | | | | | | | | -0.096 ± 0.101 | | -0.170 ± 0.234 | | | | | | 0.362 | | | | 0.462 | | | | 0.988 | |  |  |
|  | | | Striatum | | | 0.000 ± 0.264 | | | | | | 0.191 ± 0.169 | | | | | | | | | | | | 0.165 ± 0.180 | | 0.213 ± 0.178 | | | | | | 0.264 | | | | 0.158 | | | | 0.386 | |  |  |
|  | | | Thalamus | | | 0.000 ± 0.304 | | | | | | 0.084 ± 0.220 | | | | | | | | | | | | -0.109 ± 0.160 | | -0.053 ± 0.248 | | | | | | 0.219 | | | | 0.478 | | | | 0.891 | |  |  |
| *COL3A1* | | | GM | | | 0.000 ± 0.311 | | | | | | -0.159 ± 0.652 | | | | | | | | | | | | 0.418 ± 1.982 | | 0.152 ± 0.641 | | | | | | 0.510 | | | | 0.700 | | | | 0.922 | |  |  |
|  | | | WM | | | 0.000 ± 0.227 | | | | | | -0.599 ± 1.489 | | | | | | | | | | | | 0.044 ± 0.252 | | -0.331 ± 0.146 | | | | | | 0.687 | | | | 0.218 | | | | 0.772 | |  |  |
|  | | | Hipp | | | 0.000 ± 0.289 | | | | | | -0.110 ± 0.407 | | | | | | | | | | | | -0.136 ± 0.118 | | -0.055 ± 0.101 | | | | | | 0.714 | | | | 0.897 | | | | 0.396 | |  |  |
|  | | | Striatum | | | 0.000 ± 0.132 | | | | | | -0.029 ± 0.100 | | | | | | | | | | | | -0.186 ± 0.165 | | 0.104 ± 0.324 | | | | | | 0.741 | | | | 0.110 | | | | 0.054 | |  |  |
|  | | | Thalamus | | | 0.000 ± 0.129 | | | | | | -0.132 ± 0.299 | | | | | | | | | | | | -0.074 ± 0.208 | | 0.005 ± 0.155 | | | | | | 0.744 | | | | 0.784 | | | | 0.282 | |  |  |
| *PPAR**γ* | | | GM | | | 0.000 ± 0.065 | | | | | | 0.004 ± 0.128 | | | | | | | | | | | | -0.103 ± 0.123 | | -0.057 ± 0.089 | | | | | | 0.134 | | | | 0.635 | | | | 0.693 | |  |  |
|  | | | WM | | | 0.000 ± 0.188 | | | | | | -0.358 ± 0.223 | | | | | | | | | | | | -0.026 ± 0.264 | | -0.097 ± 0.172 | | | | | | 0.273 | | | | 0.054 | | | | 0.185 | |  |  |
|  | | | Hipp | | | 0.000 ± 0.142 | | | | | | -0.009 ± 0.166 | | | | | | | | | | | | 0.039 ± 0.140 | | 0.069 ± 0.137 | | | | | | 0.360 | | | | 0.866 | | | | 0.756 | |  |  |
|  | | | Striatum | | | **0.000** **± 0.295** | | | | | | **0.306 ± 0.078^&^** | | | | | | | | | | | | 0.203 ± 0.177 | | 0.174 ± 0.143 | | | | | | 0.640 | | | | 0.080 | | | | **0.037*** | |  |  |
|  | | | Thalamus | | | 0.000 ± 0.111 | | | | | | -0.011 ± 0.168 | | | | | | | | | | | | -0.207 ± 0.250 | | -0.143 ± 0.154 | | | | | | **0.050*** | | | | 0.747 | | | | 0.650 | |  |  |
| **Apoptosis related** | | | | | | | | | | | | | | | | | | | | | | | | | | | | | | | | | | | | | | | | |  |  |  |
| **Gene** | **Brain Region** | | | | | | | | | **Group** | | | | | | | | | | | | | | | | | | | | | | **2-Way ANOVA** | | | | | | | | |  |  |  |
|  |  |  |  |  |  |  |  |  |  | **SalCon** | | | | | | **CrCon** | | | | | | | **SalUCO** | | | | | **CrUCO** | | | | ***P_UCO_*** | | | | | | ***P_TREAT_*** | | ***P_INT_*** |  |  |  |
| *CAS3* | Striatum | | | | | | | 0.000 ± 0.157 | | | | | | 0.234 ± 0.172 | | | | | | | | | 0.189 ± 0.191 | | | | | 0.113 ± 0.214 | | | | 0.662 | | | | | | 0.313 | | 0.056 |  |  |  |
|  | Thalamus | | | | | | | 0.000 ± 0.180 | | | | | | 0.421 ± 1.054 | | | | | | | | | -0.327 ± 0.798 | | | | | 0.110 ± 0.353 | | | | 0.434 | | | | | | 0.297 | | 0.984 |  |  |  |
| *BAX* | Striatum | | | | | | | 0.000 ± 0.117 | | | | | | 0.104 ± 0.044 | | | | | | | | | 0.049 ± 0.105 | | | | | 0.037 ± 0.128 | | | | 0.834 | | | | | | 0.275 | | 0.177 |  |  |  |
|  | Thalamus | | | | | | | 0.000 ± 0.086 | | | | | | 0.045 ± 0.155 | | | | | | | | | -0.068 ± 0.109 | | | | | -0.079 ± 0.063 | | | | 0.069 | | | | | | 0.736 | | 0.584 |  |  |  |
| *BAK1* | Striatum | | | | | | | 0.000 ± 0.260 | | | | | | 0.240 ± 0.115 | | | | | | | | | 0.243 ± 0.141 | | | | | 0.014 ± 0.213 | | | | 0.913 | | | | | | 0.950 | | **0.007*** |  |  |  |
|  | Thalamus | | | | | | | 0.000 ± 0.177 | | | | | | -0.052 ± 0.237 | | | | | | | | | 0.033 ± 0.181 | | | | | -0.129 ± 0.116 | | | | 0.795 | | | | | | 0.216 | | 0.520 |  |  |  |
| *BCL-2* | Striatum | | | | | | | 0.000 ± 0.067 | | | | | | 0.243 ± 0.285 | | | | | | | | | -0.159 ± 0.515 | | | | | 0.249 ± 0.143 | | | | 0.582 | | | | | | **0.028*** | | 0.555 |  |  |  |
|  | Thalamus | | | | | | | 0.000 ± 0.243 | | | | | | 0.063 ± 0.545 | | | | | | | | | -0.020 ± 0.257 | | | | | 0.41 ± 0.309 | | | | 0.322 | | | | | | 0.142 | | 0.267 |  |  |  |
| *BECN1* | Striatum | | | | | | | 0.000 ± 0.067 | | | | | | 0.069 ± 0.075 | | | | | | | | | 0.017 ± 0.245 | | | | | 0.091 ± 0.139 | | | | 0.769 | | | | | | 0.284 | | 0.977 |  |  |  |
|  | Thalamus | | | | | | | 0.000 ± 0.124 | | | | | | -0.069 ± 0.258 | | | | | | | | | -0.209 ± 0.186 | | | | | -0.100 ± 0.119 | | | | 0.162 | | | | | | 0.811 | | 0.296 |  |  |  |
| **Inflammatory related** | | | | | | | | | | | | | | | | | | | | | | | | | | | | | | | | | | | | | | | | |  |  |  |
| **Gene** | | **Brain Region** | | | | | | **Group** | | | | | | | | | | | | | | | | | | | | | | | | **2-Way ANOVA** | | | | | | | | |  |  |  |
|  |  |  |  |  |  |  |  | **SalCon** | | | | | | | | | | **CrCon** | | | | **SalUCO** | | | | | | | | **CrUCO** | | ***P_UCO_*** | | | | | | ***P_TREAT_*** | | ***P_INT_*** |  |  |  |
| *CCL2* | | GM | | | | | | 0.000 ± 0.042 | | | | | | | | | | 0.071 ± 0.176 | | | | 0.228 ± 0.197 | | | | | | | | 0.039 ± 0.146 | | 0.188 | | | | | | 0.419 | | 0.085 |  |  |  |
|  | | WM | | | | | | 0.000 ± 0.262 | | | | | | | | | | -0.241 ± 0.248 | | | | -0.116 ± 0.343 | | | | | | | | -0.034 ± 0.076 | | 0.727 | | | | | | 0.541 | | 0.220 |  |  |  |
|  | | Hipp | | | | | | 0.000 ± 0.407 | | | | | | | | | | 0.651 ± 0.997 | | | | 0.500 ± 0.653 | | | | | | | | 0.697 ± 0.864 | | 0.409 | | | | | | 0.205 | | 0.490 |  |  |  |
|  | | Striatum | | | | | | 0.000 ± 0.220 | | | | | | | | | | 0.364 ± 0.520 | | | | 0.191 ± 0.451 | | | | | | | | 0.261 ± 0.470 | | 0.814 | | | | | | 0.250 | | 0.432 |  |  |  |
|  | | Thalamus | | | | | | 0.000 ± 0.208 | | | | | | | | | | 0.188 ± 0.593 | | | | -0.139 ± 0.293 | | | | | | | | 0.193 ± 0.441 | | 0.770 | | | | | | 0.154 | | 0.748 |  |  |  |
| *TNFa* | | GM | | | | | | 0.000 ± 0.093 | | | | | | | | | | 0.252 ± 0.228 | | | | -0.012 ± 0.429 | | | | | | | | 0.505 ± 0.180 | | 0.447 | | | | | | **0.028*** | | 0.404 |  |  |  |
|  | | WM | | | | | | 0.000 ± 0.245 | | | | | | | | | | -0.016 ± 0.296 | | | | -0.161 ± 0.189 | | | | | | | | -0.022 ± 0.166 | | 0.466 | | | | | | 0.591 | | 0.498 |  |  |  |
|  | | Hipp | | | | | | 0.000 ± 0.502 | | | | | | | | | | 0.326 ± 0.567 | | | | -0.035 ± 0.325 | | | | | | | | 0.108 ± 0.462 | | 0.547 | | | | | | 0.271 | | 0.664 |  |  |  |
|  | | Striatum | | | | | | 0.000 ± 0.263 | | | | | | | | | | 0.422 ± 0.356 | | | | 0.012 ± 0.312 | | | | | | | | 0.230 ± 0.549 | | 0.579 | | | | | | 0.059 | | 0.531 |  |  |  |
|  | | Thalamus | | | | | | 0.000 ± 0.282 | | | | | | | | | | 0.107 ± 0.220 | | | | -0.549 ± 0.391 | | | | | | | | -0.021 ± 0.644 | | 0.241 | | | | | | 0.182 | | 0.485 |  |  |  |
| *IL-1β* | | GM | | | | | | 0.000 ± 0.038 | | | | | | | | | | -0.009 ± 0.105 | | | | -0.085 ± 0.094 | | | | | | | | 0.063 ± 0.059 | | 0.873 | | | | | | 0.091 | | 0.057 |  |  |  |
|  | | WM | | | | | | 0.000 ± 0.166 | | | | | | | | | | -0.129 ± 0.116 | | | | -0.074 ± 0.187 | | | | | | | | -0.067 ± 0.109 | | 0.934 | | | | | | 0.406 | | 0.352 |  |  |  |
|  | | Hipp | | | | | | 0.000 ± 0.086 | | | | | | | | | | 0.204 ± 0.238 | | | | 0.149 ± 0.156 | | | | | | | | 0.198 ± 0.176 | | 0.344 | | | | | | 0.103 | | 0.307 |  |  |  |
|  | | Striatum | | | | | | 0.000 ± 0.113 | | | | | | | | | | 0.252 ± 0.164 | | | | 0.101 ± 0.340 | | | | | | | | 0.252 ± 0.164 | | 0.454 | | | | | | **0.026*** | | 0.741 |  |  |  |
|  | | Thalamus | | | | | | 0.000 ± 0.108 | | | | | | | | | | 0.013 ± 0.392 | | | | -0.110 ± 0.244 | | | | | | | | 0.171 ± 0.183 | | 0.843 | | | | | | 0.230 | | 0.274 |  |  |  |
| *IL-6* | | GM | | | | | | 0.000 ± 0.151 | | | | | | | | | | 0.403 ± 0.265 | | | | 0.311 ± 0.111 | | | | | | | | 0.437 ± 0.102 | | 0.092 | | | | | | **0.014*** | | 0.171 |  |  |  |
|  | | WM | | | | | | 0.000 ± 0.202 | | | | | | | | | | 0.137 ± 0.211 | | | | -0.049 ± 0.247 | | | | | | | | -0.085 ± 0.271 | | 0.217 | | | | | | 0.639 | | 0.424 |  |  |  |
|  | | Hipp | | | | | | 0.000 ± 0.138 | | | | | | | | | | -0.169 ± 0.334 | | | | -0.069 ± 0.175 | | | | | | | | -0.056 ± 0.505 | | 0.868 | | | | | | 0.561 | | 0.496 |  |  |  |
|  | | Striatum | | | | | | 0.000 ± 0.700 | | | | | | | | | | 0.396 ± 0.096 | | | | 0.407 ± 0.105 | | | | | | | | 0.348 ± 0.195 | | 0.252 | | | | | | 0.282 | | 0.152 |  |  |  |
| *CXCL8 (IL-8)* | | GM | | | | | | 0.000 ± 0.569 | | | | | | | | | | 0.282 ± 0.114 | | | | 0.432 ± 0.223 | | | | | | | | 0.163 ± 0.364 | | 0.585 | | | | | | 0.982 | | 0.346 |  |  |  |
|  |  | Striatum | | | | | | 0.000 ± 0.171 | | | | | | | | | | 0.391 ± 0.266 | | | | 0.043 ± 0.166 | | | | | | | | 0.068 ± 0.074 | | 0.614 | | | | | | 0.456 | | 0.511 |  |  |  |
|  |  | Thalamus | | | | | | 0.000 ± 0.107 | | | | | | | | | | 0.379 ± 0.250 | | | | 0.359 ± 0.176 | | | | | | | | 0.571 ± 0.166 | | 0.223 | | | | | | 0.193 | | 0.701 |  |  |  |
| *TLR3* | | GM | | | | | | 0.000 ± 0.020 | | | | | | | | | | -0.130 ± 0.031 | | | | -0.088 ± 0.067 | | | | | | | | -0.195 ± 0.055 | | 0.145 | | | | | | **0.031*** | | 0.819 |  |  |  |
|  | | WM | | | | | | 0.000 ± 0.073 | | | | | | | | | | -0.173 ± 0.119 | | | | -0.060 ± 0.060 | | | | | | | | 0.074 ± 0.046 | | 0.330 | | | | | | 0.838 | | 0.117 |  |  |  |
|  | | Hipp | | | | | | 0.000 ± 0.081 | | | | | | | | | | -0.026 ± 0.084 | | | | 0.056 ± 0.047 | | | | | | | | 0.072 ± 0.053 | | 0.271 | | | | | | 0.946 | | 0.760 |  |  |  |
|  | | Striatum | | | | | | 0.000 ± 0.052 | | | | | | | | | | 0.131 ± 0.060 | | | | 0.116 ± 0.078 | | | | | | | | 0.233 ± 0.115 | | 0.181 | | | | | | 0.130 | | 0.927 |  |  |  |
|  | | Thalamus | | | | | | 0.000 ± 0.098 | | | | | | | | | | 0.100 ± 0.098 | | | | 0.003 ± 0.061 | | | | | | | | -0.044 ± 0.163 | | 0.518 | | | | | | 0.807 | | 0.502 |  |  |  |
| *TLR4* | | GM | | | | | | 0.000 ± 0.034 | | | | | | | | | | -0.032 ± 0.047 | | | | 0.024 ± 0.073 | | | | | | | | 0.031 ± 0.049 | | 0.463 | | | | | | 0.832 | | 0.738 |  |  |  |
|  | | WM | | | | | | 0.000 ± 0.091 | | | | | | | | | | -0.098 ± 0.070 | | | | 0.062 ± 0.069 | | | | | | | | -0.101 ± 0.066 | | 0.721 | | | | | | 0.126 | | 0.692 |  |  |  |
|  | | Hipp | | | | | | 0.000 ± 0.292 | | | | | | | | | | -0.014 ± 0.468 | | | | -0.991 ± 0.228 | | | | | | | | -0.584 ± 0.659 | | 0.073 | | | | | | 0.590 | | 0.566 |  |  |  |
|  | | Striatum | | | | | | 0.000 ± 0.072 | | | | | | | | | | 0.224 ± 0.080 | | | | 0.168 ± 0.093 | | | | | | | | 0.213 ± 0.094 | | 0.384 | | | | | | 0.144 | | 0.321 |  |  |  |
|  | | Thalamus | | | | | | 0.000 ± 0.085 | | | | | | | | | | 0.153 ± 0.093 | | | | -0.120 ± 0.052 | | | | | | | | -0.067 ± 0.121 | | 0.077 | | | | | | 0.273 | | 0.590 |  |  |  |
| *PTGS1* | | GM | | | | | | 0.000 ± 0.082 | | | | | | | | | | -0.088 ± 0.085 | | | | -0.013 ± 0.091 | | | | | | | | -0.084 ± 0.045 | | 0.961 | | | | | | 0.359 | | 0.920 |  |  |  |
|  | | WM | | | | | | 0.000 ± 0.111 | | | | | | | | | | -0.247 ± 0.114 | | | | -0.001 ± 0.090 | | | | | | | | -0.020 ± 0.088 | | 0.327 | | | | | | 0.253 | | 0.325 |  |  |  |
|  | | Hipp | | | | | | 0.000 ± 0.077 | | | | | | | | | | 0.094 ± 0.112 | | | | 0.146 ± 0.066 | | | | | | | | 0.203 ± 0.075 | | 0.154 | | | | | | 0.393 | | 0.835 |  |  |  |
|  | | Striatum | | | | | | **0.000 ± 0.084** | | | | | | | | | | **0.356** **± 0.062^&^** | | | | 0.308 ± 0.072 | | | | | | | | 0.296 ± 0.137 | | 0.173 | | | | | | 0.063 | | **0.049*** |  |  |  |
|  | | Thalamus | | | | | | 0.000 ± 0.248 | | | | | | | | | | -0.171 ± 0.142 | | | | -0.347 ± 0.105 | | | | | | | | -0.335 ± 0.116 | | 0.101 | | | | | | 0.598 | | 0.545 |  |  |  |
| *PTGS2* | | GM | | | | | | 0.000 ± 0.120 | | | | | | | | | | -0.220 ± 0.088 | | | | -0.045 ± 0.166 | | | | | | | | -0.077 ± 0.032 | | 0.685 | | | | | | 0.303 | | 0.441 |  |  |  |
|  | | WM | | | | | | 0.000 ± 0.157 | | | | | | | | | | -0.418 ± 0.187 | | | | -0.181 ± 0.158 | | | | | | | | -0.295 ± 0.276 | | 0.884 | | | | | | 0.489 | | 0.445 |  |  |  |
|  | | Hipp | | | | | | 0.000 ± 0.068 | | | | | | | | | | -0.339 ± 0.111 | | | | -0.075 ± 0.116 | | | | | | | | -0.202 ± 0.222 | | 0.823 | | | | | | 0.106 | | 0.448 |  |  |  |
|  | | Striatum | | | | | | 0.000 ± 0.104 | | | | | | | | | | 0.431 ± 0.163 | | | | 0.298 ± 0.175 | | | | | | | | 0.175 ± 0.172 | | 0.898 | | | | | | 0.364 | | 0.111 |  |  |  |
|  | | Thalamus | | | | | | 0.000 ± 0.211 | | | | | | | | | | 0.116 ± 0.142 | | | | 0.056 ± 0.185 | | | | | | | | -0.018 ± 0.185 | | 0.833 | | | | | | 0.910 | | 0.611 |  |  |  |
| **Metabolism related** | | | | | | | | | | | | | | | | | | | | | | | | | | | | | | | | | | | | | | | | | | | |
| **Gene** | | | | | **Brain Region** | | | | **Group** | | | | | | | | | | | | | | | | | | | | | | | **2-Way ANOVA** | | | | | | | | | | | |
|  |  |  |  |  |  |  |  |  | **SalCon** | | | | | | **CrCon** | | | | | **SalUCO** | | | | | | | **CrUCO** | | | | | ***P_UCO_*** | | | ***P_TREAT_*** | | | | | ***P_INT_*** | | | |
| *SLC2A5* | | | | | GM | | | | 0.000 ± 0.097 | | | | | | 0.166 ± 0.062 | | | | | 0.322 ± 0.201 | | | | | | | 0.134 ± 0.088 | | | | | 0.324 | | | 0.942 | | | | | 0.232 | | | |
|  | | | | | WM | | | | 0.000 ± 0.058 | | | | | | -0.101 ± 0.086 | | | | | -0.0266 ± 0.043 | | | | | | | -0.137 ± 0.037 | | | | | 0.654 | | | 0.139 | | | | | 0.947 | | | |
|  | | | | | Hipp | | | | 0.000 ± 0.149 | | | | | | 0.305 ± 0.228 | | | | | 0.321 ± 0.144 | | | | | | | 0.406 ± 0.187 | | | | | 0.261 | | | 0.299 | | | | | 0.556 | | | |
|  | | | | | Striatum | | | | 0.000 ± 0.102 | | | | | | 0.200 ± 0.049 | | | | | -0.001 ± 0.256 | | | | | | | 0.283 ± 0.055 | | | | | 0.807 | | | 0.158 | | | | | 0.802 | | | |
|  | | | | | Thalamus | | | | 0.000 ± 0.056 | | | | | | -0.243 ± 0.372 | | | | | -0.302 ± 0.122 | | | | | | | -0.069 ± 0.151 | | | | | 0.768 | | | 0.982 | | | | | 0.278 | | | |
| *SLC6A8* | | | | | Striatum | | | | 0.000 ± 0.193 | | | | | | 0.423 ± 0.040 | | | | | 0.176 ± 0.246 | | | | | | | 0.288 ± 0.037 | | | | | 0.906 | | | 0.132 | | | | | 0.373 | | | |
|  | | | | | Thalamus | | | | 0.000 ± 0.077 | | | | | | -0.140 ± 0.105 | | | | | -0.470 ± 0.210 | | | | | | | -0.208 ± 0.142 | | | | | 0.126 | | | 0.721 | | | | | 0.247 | | | |
| *SLC5A7* | | | | | GM | | | | 0.000 ± 0.188 | | | | | | 0.445 ± 0.516 | | | | | -0.008 ± 0.259 | | | | | | | 0.053 ± 0.262 | | | | | 0.584 | | | 0.490 | | | | | 0.599 | | | |
|  | | | | | WM | | | | 0.000 ± 0.420 | | | | | | -0.091 ± 0.174 | | | | | 0.199 ± 0.475 | | | | | | | -0.105 ± 0.559 | | | | | 0.829 | | | 0.647 | | | | | 0.804 | | | |
|  | | | | | Hipp | | | | 0.000 ± 0.281 | | | | | | 1.093 ± 0.237 | | | | | 0.202 ± 0.127 | | | | | | | 0.283 ± 0.465 | | | | | 0.281 | | | **0.047*** | | | | | 0.082 | | | |
|  | | | | | Striatum | | | | 0.000 ± 0.152 | | | | | | -0.101 ± 0.271 | | | | | 0.300 ± 0.202 | | | | | | | 0.074 ± 0.112 | | | | | 0.287 | | | 0.460 | | | | | 0.775 | | | |
|  | | | | | Thalamus | | | | 0.000 ± 0.137 | | | | | | 0.331 ± 0.295 | | | | | -0.617 ± 0.103 | | | | | | | -0.241 ± 0.234 | | | | | **0.009*** | | | 0.099 | | | | | 0.914 | | | |
| *ERRα* | | | | | Striatum | | | | 0.000 ± 0.252 | | | | | | 0.210 ± 0.057 | | | | | -0.003 ± 0.245 | | | | | | | 0.097 ± 0.095 | | | | | 0.759 | | | 0.417 | | | | | 0.772 | | | |
|  | | | | | Thalamus | | | | 0.000 ± 0.141 | | | | | | 0.061 ± 0.052 | | | | | 0.072 ± 0.077 | | | | | | | -0.085 ± 0.216 | | | | | 0.799 | | | 0.741 | | | | | 0.457 | | | |
| *CKMT* | | | | | Striatum | | | | 0.000 ± 0.216 | | | | | | 0.434 ± 0.056 | | | | | 0.254 ± 0.088 | | | | | | | 0.587 ± 0.239 | | | | | 0.182 | | | **0.017*** | | | | | 0.735 | | | |
|  | | | | | Thalamus | | | | 0.000 ± 0.165 | | | | | | 0.074 ± 0.218 | | | | | -0.135 ± 0.122 | | | | | | | -0.198 ± 0.088 | | | | | 0.230 | | | 0.973 | | | | | 0.681 | | | |
| *FASN* | | | | | GM | | | | 0.000 ± 0.040 | | | | | | 0.006 ± 0.035 | | | | | -0.173 ± 0.015 | | | | | | | -0.095 ± 0.069 | | | | | **0.005*** | | | 0.342 | | | | | 0.411 | | | |
|  | | | | | WM | | | | 0.000 ± 0.047 | | | | | | -0.185 ± 0.055 | | | | | 0.006 ± 0.069 | | | | | | | -0.089 ± 0.072 | | | | | 0.460 | | | 0.055 | | | | | 0.510 | | | |
|  | | | | | Hipp | | | | 0.000 ± 0.037 | | | | | | -0.057 ± 0.049 | | | | | -0.022 ± 0.039 | | | | | | | -0.080 ± 0.069 | | | | | 0.658 | | | 0.258 | | | | | 0.992 | | | |
|  | | | | | Striatum | | | | 0.000 ± 0.137 | | | | | | 0.302 ± 0.026 | | | | | 0.232 ± 0.099 | | | | | | | 0.218 ± 0.037 | | | | | 0.393 | | | 0.106 | | | | | 0.079 | | | |
|  | | | | | Thalamus | | | | 0.000 ± 0.058 | | | | | | -0.055 ± 0.046 | | | | | -0.222 ± 0.082 | | | | | | | -0.121 ± 0.074 | | | | | 0.057 | | | 0.749 | | | | | 0.287 | | | |
| *PDK1* | | | | | GM | | | | 0.000 ± 0.347 | | | | | | -0.314 ± 0.216 | | | | | 0.435 ± 0.715 | | | | | | | 0.188 ± 0.292 | | | | | 0.441 | | | 0.699 | | | | | 0.901 | | | |
|  | | | | | WM | | | | 0.000 ± 0.051 | | | | | | -0.154 ± 0.084 | | | | | -0.010 ± 0.053 | | | | | | | 0.017 ± 0.073 | | | | | 0.275 | | | 0.386 | | | | | 0.222 | | | |
|  | | | | | Hipp | | | | 0.000 ± 0.007 | | | | | | -0.098 ± 0.053 | | | | | -0.105 ± 0.028 | | | | | | | -0.150 ± 0.094 | | | | | 0.151 | | | 0.190 | | | | | 0.616 | | | |
|  | | | | | Striatum | | | | 0.000 ± 0.108 | | | | | | 0.102 ± 0.066 | | | | | 0.029 ± 0.070 | | | | | | | 0.094 ± 0.100 | | | | | 0.901 | | | 0.334 | | | | | 0.827 | | | |
|  | | | | | Thalamus | | | | 0.000 ± 0.100 | | | | | | -0.324 ± 0.162 | | | | | -0.268 ± 0.043 | | | | | | | -0.290 ± 0.095 | | | | | 0.348 | | | 0.162 | | | | | 0.220 | | | |
| *PRKAA1* | | | | | Striatum | | | | 0.000 ± 0.099 | | | | | | 0.123 ± 0.075 | | | | | 0.087 ± 0.072 | | | | | | | 0.104 ± 0.099 | | | | | 0.688 | | | 0.418 | | | | | 0.544 | | | |
|  | | | | | Thalamus | | | | 0.000 ± 0.118 | | | | | | -0.304 ± 0.160 | | | | | -0.219 ± 0.054 | | | | | | | -0.233 ± 0.071 | | | | | 0.543 | | | 0.199 | | | | | 0.238 | | | |
| *PRKAA2* | | | | | Striatum | | | | 0.000 ± 0.110 | | | | | | 0.243 ± 0.063 | | | | | 0.066 ± 0.139 | | | | | | | 0.150 ± 0.082 | | | | | 0.901 | | | 0.145 | | | | | 0.470 | | | |
|  | | | | | Thalamus | | | | 0.000 ± 0.070 | | | | | | -0.188 ± 0.129 | | | | | -0.279 ± 0.090 | | | | | | | -0.217 ± 0.064 | | | | | 0.167 | | | 0.560 | | | | | 0.258 | | | |
| **Mitochondria related** | | | | | | | | | | | | | | | | | | | | | | | | | | | | | | | | | | | | | | | | | | |  |
| **Gene** | | **Brain Region** | | | | | | | | | **Group** | | | | | | | | | | | | | | | | | | | | | | **2-Way ANOVA** | | | | | | | | | |  |
|  |  |  |  |  |  |  |  |  |  |  | **SalCon** | | | | | | **CrCon** | | | | | | **SalUCO** | | | | | | **CrUCO** | | | | ***P_UCO_*** | | | | ***P_TREAT_*** | | | ***P_INT_*** | | |  |
| *MFN1* | | Striatum | | | | | | | | | 0.000 ± 0.121 | | | | | | 0.268 ± 0.056 | | | | | | 0.180 ± 0.081 | | | | | | 0.156 ± 0.071 | | | | 0.690 | | | | 0.157 | | | 0.094 | | |  |
|  | | Thalamus | | | | | | | | | 0.000 ± 0.107 | | | | | | -0.238 ± 0.070 | | | | | | -0.207 ± 0.092 | | | | | | -0.231 ± 0.056 | | | | 0.259 | | | | 0.143 | | | 0.231 | | |  |
| *MFN2* | | Striatum | | | | | | | | | 0.000 ± 0.172 | | | | | | 0.258 ± 0.051 | | | | | | 0.116 ± 0.139 | | | | | | 0.148 ± 0.079 | | | | 0.980 | | | | 0.235 | | | 0.354 | | |  |
|  | | Thalamus | | | | | | | | | 0.000 ± 0.028 | | | | | | -0.202 ± 0.128 | | | | | | -0.365 ± 0.131 | | | | | | -0.169 ± 0.079 | | | | 0.191 | | | | 0.982 | | | 0.120 | | |  |
| *FIS1* | | Striatum | | | | | | | | | 0.000 ± 0.089 | | | | | | -0.135 ± 0.027 | | | | | | -0.092 ± 0.070 | | | | | | -0.090 ± 0.080 | | | | 0.733 | | | | 0.330 | | | 0.319 | | |  |
|  | | Thalamus | | | | | | | | | 0.000 ± 0.163 | | | | | | 0.024 ± 0.130 | | | | | | -0.070 ± 0.169 | | | | | | 0.174 ± 0.078 | | | | 0.793 | | | | 0.384 | | | 0.473 | | |  |
| *DNM1L* | | Striatum | | | | | | | | | 0.000 ± 0.122 | | | | | | 0.225 ± 0.056 | | | | | | 0.139 ± 0.085 | | | | | | 0.100 ± 0.088 | | | | 0.941 | | | | 0.300 | | | 0.145 | | |  |
|  | | Thalamus | | | | | | | | | 0.000 ± 0.060 | | | | | | -0.162 ± 0.059 | | | | | | -0.218 ± 0.065 | | | | | | -0.193 ± 0.081 | | | | 0.089 | | | | 0.336 | | | 0.193 | | |  |
| *OPA1* | | Striatum | | | | | | | | | 0.000 ± 0.106 | | | | | | 0.208 ± 0.058 | | | | | | 0.086 ± 0.084 | | | | | | 0.119 ± 0.065 | | | | 0.985 | | | | 0.147 | | | 0.285 | | |  |
|  | | Thalamus | | | | | | | | | 0.000 ± 0.045 | | | | | | -0.127 ± 0.079 | | | | | | -0.249 ± 0.060 | | | | | | -0.164 ± 0.089 | | | | 0.075 | | | | 0.788 | | | 0.179 | | |  |
| *MFF* | | Striatum | | | | | | | | | 0.000 ± 0.085 | | | | | | 0.134 ± 0.024 | | | | | | 0.026 ± 0.118 | | | | | | 0.058 ± 0.065 | | | | 0.773 | | | | 0.339 | | | 0.555 | | |  |
|  | | Thalamus | | | | | | | | | 0.000 ± 0.038 | | | | | | -0.027 ± 0.033 | | | | | | -0.150 ± 0.070 | | | | | | -0.124 ± 0.040 | | | | **0.034*** | | | | 0.990 | | | 0.630 | | |  |
| *B2M* | | Striatum | | | | | | | | | 0.000 ± 0.050 | | | | | | 0.061 ± 0.082 | | | | | | 0.017 ± 0.059 | | | | | | 0.171 ± 0.084 | | | | 0.396 | | | | 0.156 | | | 0.535 | | |  |
|  | | Thalamus | | | | | | | | | 0.000 ± 0.056 | | | | | | 0.017 ± 0.186 | | | | | | 0.051 ± 0.040 | | | | | | 0.108 ± 0.110 | | | | 0.595 | | | | 0.782 | | | 0.882 | | |  |
| *TRMT11* | | Striatum | | | | | | | | | 0.000 ± 0.065 | | | | | | 0.129 ± 0.0256 | | | | | | 0.136 ± 0.064 | | | | | | 0.094 ± 0.053 | | | | 0.363 | | | | 0.434 | | | 0.130 | | |  |
|  | | Thalamus | | | | | | | | | 0.000 ± 0.079 | | | | | | 0.147 ± 0.096 | | | | | | -0.009 ± 0.051 | | | | | | -0.028 ± 0.062 | | | | 0.262 | | | | 0.429 | | | 0.309 | | |  |
| *NDUFBB (CII)* | | Striatum | | | | | | | | | 0.000 ± 0.036 | | | | | | -0.061 ± 0.056 | | | | | | -0.034 ± 0.031 | | | | | | -0.091 ± 0.047 | | | | 0.468 | | | | 0.222 | | | 0.932 | | |  |
|  |  | Thalamus | | | | | | | | | 0.000 ± 0.104 | | | | | | -0.110 ± 0.082 | | | | | | -0.071 ± 0.065 | | | | | | -0.183 ± 0.053 | | | | 0.372 | | | | 0.176 | | | 0.991 | | |  |
| *UQCRH (CIII)* | | Striatum | | | | | | | | | 0.000 ± 0.067 | | | | | | -0.147 ± 0.031 | | | | | | -0.061 ± 0.057 | | | | | | -0.057 ± 0.041 | | | | 0.782 | | | | 0.176 | | | 0.151 | | |  |
|  |  | Thalamus | | | | | | | | | 0.000 ± 0.029 | | | | | | 0.028 ± 0.062 | | | | | | -0.139 ± 0.036 | | | | | | -0.050 ± 0.027 | | | | **0.039*** | | | | 0.243 | | | 0.533 | | |  |
| *LOC101105 (CIV)* | | Striatum | | | | | | | | | 0.000 ± 0.017 | | | | | | -0.011 ± 0.020 | | | | | | 0.006 ± 0.018 | | | | | | -0.011 ± 0.036 | | | | 0.896 | | | | 0.544 | | | 0.908 | | |  |
|  |  | Thalamus | | | | | | | | | 0.000 ± 0.023 | | | | | | 0.084 ± 0.080 | | | | | | -0.042 ± 0.029 | | | | | | 0.031 ± 0.051 | | | | 0.435 | | | | 0.201 | | | 0.926 | | |  |
| *ATP6PO (CV)* | | Striatum | | | | | | | | | 0.000 ± 0.017 | | | | | | -0.050 ± 0.026 | | | | | | -0.008 ± 0.013 | | | | | | -0.026 ± 0.019 | | | | 0.711 | | | | 0.119 | | | 0.441 | | |  |
|  |  | Thalamus | | | | | | | | | 0.000 ± 0.049 | | | | | | 0.028 ± 0.034 | | | | | | -0.058 ± 0.023 | | | | | | 0.028 ± 0.034 | | | | 0.191 | | | | 0.397 | | | 0.893 | | |  |
| *MIEF1* | | Striatum | | | | | | | | | 0.000 ± 0.153 | | | | | | 0.308 ± 0.049 | | | | | | 0.165 ± 0.134 | | | | | | 0.224 ± 0.064 | | | | 0.714 | | | | 0.110 | | | 0.271 | | |  |
|  | | Thalamus | | | | | | | | | 0.000 ± 0.060 | | | | | | -0.244 ± 0.144 | | | | | | -0.322 ± 0.127 | | | | | | -0.193 ± 0.091 | | | | 0.311 | | | | 0.664 | | | 0.169 | | |  |
| *CRLS1* | | Striatum | | | | | | | | | 0.000 ± 0.058 | | | | | | 0.143 ± 0.032 | | | | | | 0.130 ± 0.062 | | | | | | 0.094 ± 0.045 | | | | 0.443 | | | | 0.312 | | | 0.099 | | |  |
|  | | Thalamus | | | | | | | | | 0.000 ± 0.078 | | | | | | -0.003 ± 0.067 | | | | | | -0.090 ± 0.047 | | | | | | -0.178 ± 0.065 | | | | 0.057 | | | | 0.492 | | | 0.525 | | |  |
| *TFAM* | | Striatum | | | | | | | | | 0.000 ± 0.121 | | | | | | -0.109 ± 0.182 | | | | | | 0.262 ± 0.276 | | | | | | 0.053 ± 0.234 | | | | 0.418 | | | | 0.543 | | | 0.848 | | |  |
|  | | Thalamus | | | | | | | | | 0.000 ± 0.167 | | | | | | 0.050 ± 0.087 | | | | | | -0.180 ± 0.061 | | | | | | -0.250 ± 0.162 | | | | **0.049*** | | | | 0.931 | | | 0.604 | | |  |
| *MSTN* | | Striatum | | | | | | | | | 0.000 ± 0.097 | | | | | | 0.109 ± 0.054 | | | | | | 0.113 ± 0.060 | | | | | | 0.081 ± 0.096 | | | | 0.573 | | | | 0.611 | | | 0.355 | | |  |
|  | | Thalamus | | | | | | | | | 0.000 ± 0.079 | | | | | | 0.075 ± 0.086 | | | | | | 0.031 ± 0.053 | | | | | | -0.195 ± 0.066 | | | | 0.116 | | | | 0.312 | | | 0.052 | | |  |
| *SIRT1* | | Striatum | | | | | | | | | 0.000 ± 0.073 | | | | | | 0.137 ± 0.055 | | | | | | 0.063 ± 0.075 | | | | | | 0.090 ± 0.080 | | | | 0.913 | | | | 0.267 | | | 0.452 | | |  |
|  | | Thalamus | | | | | | | | | 0.000 ± 0.107 | | | | | | 0.028 ± 0.075 | | | | | | -0.133 ± 0.025 | | | | | | -0.158 ± 0.075 | | | | **0.036*** | | | | 0.989 | | | 0.709 | | |  |
| *SIRT3* | | Striatum | | | | | | | | | 0.000 ± 0.050 | | | | | | 0.019 ± 0.036 | | | | | | -0.128 ± 0.152 | | | | | | 0.060 ± 0.042 | | | | 0.659 | | | | 0.300 | | | 0.395 | | |  |
|  | | Thalamus | | | | | | | | | 0.000 ± 0.052 | | | | | | -0.089 ± 0.157 | | | | | | -0.343 ± 0.154 | | | | | | -0.037 ± 0.065 | | | | 0.326 | | | | 0.462 | | | 0.187 | | |  |
| *CPT1A* | | Striatum | | | | | | | | | 0.000 ± 0.149 | | | | | | 0.184 ± 0.065 | | | | | | -0.008 ± 0.164 | | | | | | 0.138 ± 0.107 | | | | 0.839 | | | | 0.219 | | | 0.884 | | |  |
|  | | Thalamus | | | | | | | | | 0.000 ± 0.091 | | | | | | -0.080 ± 0.097 | | | | | | -0.268 ± 0.088 | | | | | | -0.180 ± 0.071 | | | | 0.066 | | | | 0.970 | | | 0.387 | | |  |
| **Oxidative stress related** | | | | | | | | | | | | | | | | | | | | | | | | | | | | | | | | | | | | | | | | | | | |
| **Gene** | | | | | **Brain Region** | | | | **Group** | | | | | | | | | | | | | | | | | | | | | | | **2-Way ANOVA** | | | | | | | | | | | |
|  |  |  |  |  |  |  |  |  | **SalCon** | | | | | | **CrCon** | | | | | | **SalUCO** | | | | | | **CrUCO** | | | | | ***P_UCO_*** | | | ***P_TREAT_*** | | | | | ***P_INT_*** | | | |
| *MPO* | | | | | GM | | | | 0.000 ± 0.139 | | | | | | 0.163 ± 0.162 | | | | | | -0.176 ± 0.254 | | | | | | 0.009 ± 0.179 | | | | | 0.434 | | | 0.457 | | | | | 0.993 | | | |
|  | | | | | Hipp | | | | 0.000 ± 0.213 | | | | | | -0.044 ± 0.305 | | | | | | -0.084 ± 0.145 | | | | | | 0.335 ± 0.169 | | | | | 0.496 | | | 0.389 | | | | | 0.291 | | | |
|  | | | | | Striatum | | | | 0.000 ± 0.142 | | | | | | 0.803 ± 0.152 | | | | | | 0.266 ± 0.123 | | | | | | 0.494 ± 0.110 | | | | | 0.886 | | | **0.003*** | | | | | 0.066 | | | |
|  | | | | | Thalamus | | | | 0.000 ± 0.278 | | | | | | 0.332 ± 0.241 | | | | | | -0.118 ± 0.406 | | | | | | 0.359 ± 0.164 | | | | | 0.876 | | | 0.183 | | | | | 0.804 | | | |
| *NOX1* | | | | | WM | | | | 0.000 ± 0.250 | | | | | | -0.177 ± 0.327 | | | | | | 0.189 ± 0.148 | | | | | | 0.159 ± 0.192 | | | | | 0.310 | | | 0.683 | | | | | 0.771 | | | |
|  | | | | | Hipp | | | | **0.000 ± 0.209** | | | | | | **-0.660 ± 0.077^&^** | | | | | | -0.327 ± 0.075 | | | | | | -0.334 ± 0.137 | | | | | 0.993 | | | **0.015*** | | | | | **0.016*** | | | |
|  | | | | | Striatum | | | | 0.000 ± 0.126 | | | | | | 0.263 ± 0.184 | | | | | | 0.181 ± 0.242 | | | | | | 0.599 ± 0.116 | | | | | 0.258 | | | 0.155 | | | | | 0.716 | | | |
| *CYBB* | | | | | GM | | | | 0.000 ± 0.042 | | | | | | -0.044 ± 0.048 | | | | | | -0.164 ± 0.106 | | | | | | -0.044 ± 0.044 | | | | | 0.272 | | | 0.610 | | | | | 0.271 | | | |
|  | | | | | WM | | | | 0.000 ± 0.097 | | | | | | -0.238 ± 0.083 | | | | | | -0.018 ± 0.085 | | | | | | -0.038 ± 0.080 | | | | | 0.354 | | | 0.193 | | | | | 0.267 | | | |
|  | | | | | Hipp | | | | 0.000 ± 0.044 | | | | | | 0.138 ± 0.234 | | | | | | 0.071 ± 0.067 | | | | | | 0.079 ± 0.167 | | | | | 0.968 | | | 0.634 | | | | | 0.671 | | | |
|  | | | | | Striatum | | | | 0.000 ± 0.151 | | | | | | 0.273 ± 0.052 | | | | | | 0.124 ± 0.110 | | | | | | 0.139 ± 0.123 | | | | | 0.966 | | | 0.200 | | | | | 0.251 | | | |
|  | | | | | Thalamus | | | | 0.000 ± 0.152 | | | | | | -0.078 ± 0.056 | | | | | | -0.396 ± 0.159 | | | | | | -0.150 ± 0.090 | | | | | 0.087 | | | 0.526 | | | | | 0.227 | | | |
| *GPX1* | | | | | GM | | | | 0.000 ± 0.056 | | | | | | -0.034 ± 0.036 | | | | | | -0.027 ± 0.038 | | | | | | 0.062 ± 0.021 | | | | | 0.382 | | | 0.492 | | | | | 0.131 | | | |
|  | | | | | WM | | | | 0.000 ± 0.062 | | | | | | -0.042 ± 0.028 | | | | | | -0.022 ± 0.035 | | | | | | -0.060 ± 0.035 | | | | | 0.655 | | | 0.379 | | | | | 0.968 | | | |
|  | | | | | Hipp | | | | 0.000 ± 0.034 | | | | | | 0.118 ± 0.095 | | | | | | 0.044 ± 0.059 | | | | | | 0.114 ± 0.050 | | | | | 0.774 | | | 0.181 | | | | | 0.723 | | | |
|  | | | | | Striatum | | | | 0.000 ± 0.050 | | | | | | 0.132 ± 0.034 | | | | | | 0.001 ± 0.092 | | | | | | 0.109 ± 0.046 | | | | | 0.863 | | | 0.081 | | | | | 0.849 | | | |
|  | | | | | Thalamus | | | | 0.000 ± 0.052 | | | | | | -0.052 ± 0.096 | | | | | | -0.020 ± 0.040 | | | | | | 0.087 ± 0.080 | | | | | 0.450 | | | 0.727 | | | | | 0.319 | | | |
| *CAT* | | | | | GM | | | | 0.000 ± 0.017 | | | | | | -0.056 ± 0.045 | | | | | | -0.038 ± 0.086 | | | | | | -0.063 ± 0.034 | | | | | 0.725 | | | 0.535 | | | | | 0.815 | | | |
|  | | | | | WM | | | | 0.000 ± 0.055 | | | | | | -0.141 ± 0.044 | | | | | | -0.021 ± 0.053 | | | | | | -0.012 ± 0.070 | | | | | 0.360 | | | 0.266 | | | | | 0.207 | | | |
|  | | | | | Hipp | | | | 0.000 ± 0.029 | | | | | | -0.001 ± 0.038 | | | | | | 0.071 ± 0.027 | | | | | | -0.032 ± 0.062 | | | | | 0.622 | | | 0.206 | | | | | 0.213 | | | |
|  | | | | | Striatum | | | | 0.000 ± 0.083 | | | | | | 0.138 ± 0.048 | | | | | | 0.043 ± 0.099 | | | | | | 0.139 ± 0.067 | | | | | 0.794 | | | 0.173 | | | | | 0.804 | | | |
|  | | | | | Thalamus | | | | 0.000 ± 0.059 | | | | | | -0.036 ± 0.123 | | | | | | -0.126 ± 0.073 | | | | | | -0.065 ± 0.078 | | | | | 0.419 | | | 0.896 | | | | | 0.616 | | | |
| *SOD2* | | | | | GM | | | | 0.000 ± 0.020 | | | | | | -0.090 ± 0.048 | | | | | | 0.020 ± 0.172 | | | | | | 0.037 ± 0.078 | | | | | 0.543 | | | 0.761 | | | | | 0.659 | | | |
|  | | | | | WM | | | | 0.000 ± 0.073 | | | | | | -0.132 ± 0.039 | | | | | | -0.021 ± 0.068 | | | | | | -0.004 ± 0.050 | | | | | 0.436 | | | 0.404 | | | | | 0.280 | | | |
|  | | | | | Hipp | | | | 0.000 ± 0.053 | | | | | | 0.089 ± 0.037 | | | | | | 0.067 ± 0.044 | | | | | | 0.109 ± 0.039 | | | | | 0.341 | | | 0.157 | | | | | 0.597 | | | |
|  | | | | | Striatum | | | | 0.000 ± 0.053 | | | | | | 0.161 ± 0.034 | | | | | | 0.056 ± 0.111 | | | | | | 0.171 ± 0.059 | | | | | 0.676 | | | 0.089 | | | | | 0.770 | | | |
|  | | | | | Thalamus | | | | 0.000 ± 0.027 | | | | | | -0.070 ± 0.120 | | | | | | -0.175 ± 0.068 | | | | | | -0.005 ± 0.096 | | | | | 0.580 | | | 0.613 | | | | | 0.235 | | | |
| *SOD3* | | | | | GM | | | | 0.000 ± 0.124 | | | | | | -0.113 ± 0.079 | | | | | | 0.004 ± 0.067 | | | | | | 0.068 ± 0.093 | | | | | 0.324 | | | 0.792 | | | | | 0.433 | | | |
|  | | | | | WM | | | | 0.000 ± 0.123 | | | | | | -0.147 ± 0.114 | | | | | | 0.128 ± 0.194 | | | | | | -0.136 ± 0.251 | | | | | 0.713 | | | 0.285 | | | | | 0.756 | | | |
|  | | | | | Hipp | | | | 0.000 ± 0.094 | | | | | | -0.046 ± 0.092 | | | | | | 0.098 ± 0.087 | | | | | | 0.104 ± 0.069 | | | | | 0.193 | | | 0.829 | | | | | 0.776 | | | |
|  | | | | | Striatum | | | | 0.000 ± 0.174 | | | | | | 0.231 ± 0.100 | | | | | | 0.268 ± 0.131 | | | | | | 0.231 ± 0.100 | | | | | 0.370 | | | 0.511 | | | | | 0.385 | | | |
|  | | | | | Thalamus | | | | 0.000 ± 0.158 | | | | | | 0.293 ± 0.143 | | | | | | 0.018 ± 0.084 | | | | | | -0.392 ± 0.306 | | | | | 0.082 | | | 0.749 | | | | | 0.069 | | | |
| *NOS1* | | | | | GM | | | | 0.000 ± 0.297 | | | | | | -0.372 ± 0.253 | | | | | | 0.364 ± 0.886 | | | | | | -0.040 ± 0.188 | | | | | 0.518 | | | 0.472 | | | | | 0.976 | | | |
|  | | | | | WM | | | | 0.000 ± 0.109 | | | | | | -0.478 ± 0.164 | | | | | | -0.038 ± 0.131 | | | | | | -0.173 ± 0.277 | | | | | 0.434 | | | 0.083 | | | | | 0.318 | | | |
|  | | | | | Hipp | | | | 0.000 ± 0.107 | | | | | | -0.065 ± 0.236 | | | | | | 0.049 ± 0.079 | | | | | | -0.027 ± 0.214 | | | | | 0.800 | | | 0.681 | | | | | 0.974 | | | |
|  | | | | | Striatum | | | | 0.000 ± 0.214 | | | | | | 0.354 ± 0.058 | | | | | | 0.090 ± 0.270 | | | | | | 0.129 ± 0.130 | | | | | 0.733 | | | 0.326 | | | | | 0.430 | | | |
|  | | | | | Thalamus | | | | 0.000 ± 0.295 | | | | | | -0.247 ± 0.261 | | | | | | -0.762 ± 0.134 | | | | | | -0.360 ± 0.195 | | | | | 0.063 | | | 0.730 | | | | | 0.158 | | | |
| *NOS2* | | | | | GM | | | | 0.000 ± 0.091 | | | | | | -0.195 ± 0.095 | | | | | | -0.322 ± 0.088 | | | | | | -0.265 ± 0.288 | | | | | 0.202 | | | 0.647 | | | | | 0.405 | | | |
|  | | | | | WM | | | | 0.000 ± 0.066 | | | | | | -0.148 ± 0.100 | | | | | | 0.089 ± 0.185 | | | | | | -0.252 ± 0.381 | | | | | 0.969 | | | 0.213 | | | | | 0.615 | | | |
|  | | | | | Hipp | | | | 0.000 ± 0.088 | | | | | | -0.136 ± 0.083 | | | | | | -0.194 ± 0.101 | | | | | | 0.060 ± 0.121 | | | | | 0.991 | | | 0.587 | | | | | 0.082 | | | |
|  | | | | | Striatum | | | | 0.000 ± 0.054 | | | | | | 0.249 ± 0.115 | | | | | | 0.099 ± 0.143 | | | | | | 0.014 ± 0.189 | | | | | 0.640 | | | 0.574 | | | | | 0.255 | | | |
|  | | | | | Thalamus | | | | 0.000 ± 0.169 | | | | | | 0.049 ± 0.038 | | | | | | 0.045 ± 0.125 | | | | | | -0.135 ± 0.210 | | | | | 0.650 | | | 0.671 | | | | | 0.461 | | | |
| *NOS3* | | | | | GM | | | | 0.000 ± 0.150 | | | | | | -0.276 ± 0.104 | | | | | | -0.142 ± 0.226 | | | | | | -0.063 ± 0.197 | | | | | 0.863 | | | 0.631 | | | | | 0.391 | | | |
|  | | | | | Striatum | | | | 0.000 ± 0.386 | | | | | | -0.139 ± 0.114 | | | | | | -0.354 ± 0.023 | | | | | | 0.037 ± 0.060 | | | | | 0.644 | | | 0.518 | | | | | 0.195 | | | |
| *GSR* | | | | | GM | | | | 0.000 ± 0.010 | | | | | | 0.019 ± 0.030 | | | | | | 0.205 ± 0.298 | | | | | | -0.014 ± 0.020 | | | | | 0.659 | | | 0.609 | | | | | 0.542 | | | |
|  | | | | | WM | | | | 0.000 ± 0.042 | | | | | | 0.296 ± 0.169 | | | | | | 0.175 ± 0.079 | | | | | | 0.144 ± 0.058 | | | | | 0.925 | | | 0.288 | | | | | 0.194 | | | |
|  | | | | | Hipp | | | | 0.000 ± 0.347 | | | | | | -0.127 ± 0.466 | | | | | | 0.050 ± 0.229 | | | | | | -0.446 ± 0.206 | | | | | 0.693 | | | 0.363 | | | | | 0.588 | | | |
|  | | | | | Striatum | | | | 0.000 ± 0.042 | | | | | | 0.004 ± 0.030 | | | | | | 0.036 ± 0.049 | | | | | | 0.028 ± 0.025 | | | | | 0.457 | | | 0.960 | | | | | 0.889 | | | |
|  | | | | | Thalamus | | | | 0.000 ± 0.138 | | | | | | -0.022 ± 0.088 | | | | | | -0.083 ± 0.088 | | | | | | -0.048 ± 0.066 | | | | | 0.579 | | | 0.945 | | | | | 0.769 | | | |
| *NFE2L2* | | | | | GM | | | | 0.000 ± 0.027 | | | | | | -0.047 ± 0.025 | | | | | | 0.176 ± 0.213 | | | | | | -0.025 ± 0.021 | | | | | 0.481 | | | 0.380 | | | | | 0.582 | | | |
|  | | | | | WM | | | | 0.000 ± 0.072 | | | | | | -0.063 ± 0.056 | | | | | | -0.037 ± 0.055 | | | | | | 0.006 ± 0.038 | | | | | 0.810 | | | 0.879 | | | | | 0.425 | | | |
|  | | | | | Hipp | | | | 0.000 ± 0.043 | | | | | | -0.018 ± 0.087 | | | | | | -0.055 ± 0.032 | | | | | | -0.050 ± 0.043 | | | | | 0.451 | | | 0.910 | | | | | 0.841 | | | |
|  | | | | | Striatum | | | | 0.000 ± 0.052 | | | | | | 0.136 ± 0.055 | | | | | | 0.118 ± 0.074 | | | | | | 0.162 ± 0.085 | | | | | 0.310 | | | 0.207 | | | | | 0.515 | | | |
|  | | | | | Thalamus | | | | 0.000 ± 0.067 | | | | | | 0.316 ± 0.104 | | | | | | 0.053 ± 0.053 | | | | | | 0.024 ± 0.099 | | | | | 0.196 | | | 0.125 | | | | | 0.067 | | | |
| **Vasculature related** | | | | | | | | | | | | | | | | | | | | | | | | | | | | | | | | | | | | | | | | | | | |
| **Gene** | | | | **Brain Region** | | | **Group** | | | | | | | | | | | | | | | | | | | | | | | | **2-Way ANOVA** | | | | | | | | | | | | |
|  |  |  |  |  |  |  | **SalCon** | | | | | | **CrCon** | | | | | | **SalUCO** | | | | | | **CrUCO** | | | | | | ***P_UCO_*** | | | ***P_TREAT_*** | | | | | ***P_INT_*** | | | | |
| *VEGFA* | | | | GM | | | 0.000 ± 0.052 | | | | | | -0.039 ± 0.078 | | | | | | -0.242 ± 0.038 | | | | | | -0.085 ± 0.061 | | | | | | **0.041*** | | | 0.380 | | | | | 0.154 | | | | |
|  | | | | WM | | | 0.000 ± 0.080 | | | | | | -0.283 ± 0.145 | | | | | | -0.036 ± 0.078 | | | | | | -0.015 ± 0.094 | | | | | | 0.327 | | | 0.269 | | | | | 0.204 | | | | |
|  | | | | Hipp | | | 0.000 ± 0.053 | | | | | | -0.079 ± 0.077 | | | | | | -0.120 ± 0.043 | | | | | | -0.094 ± 0.081 | | | | | | 0.308 | | | 0.684 | | | | | 0.428 | | | | |
|  | | | | Striatum | | | 0.000 ± 0.187 | | | | | | -0.533 ± 0.296 | | | | | | 0.251 ± 0.263 | | | | | | -0.077 ± 0.183 | | | | | | 0.191 | | | 0.115 | | | | | 0.699 | | | | |
|  | | | | Thalamus | | | 0.000 ± 0.097 | | | | | | -0.264 ± 0.236 | | | | | | -0.274 ± 0.133 | | | | | | -0.180 ± 0.083 | | | | | | 0.604 | | | 0.643 | | | | | 0.332 | | | | |
| *OCLN* | | | | GM | | | 0.000 ± 0.086 | | | | | | 0.046 ± 0.026 | | | | | | -0.039 ± 0.060 | | | | | | -0.070 ± 0.044 | | | | | | 0.160 | | | 0.886 | | | | | 0.480 | | | | |
|  | | | | WM | | | 0.000 ± 0.057 | | | | | | 0.074 ± 0.177 | | | | | | 0.052 ± 0.072 | | | | | | -0.048 ± 0.113 | | | | | | 0.787 | | | 0.918 | | | | | 0.500 | | | | |
|  | | | | Hipp | | | 0.000 ± 0.083 | | | | | | -0.185 ± 0.104 | | | | | | -0.171 ± 0.032 | | | | | | -0.034 ± 0.032 | | | | | | 0.885 | | | 0.736 | | | | | **0.033*** | | | | |
|  | | | | Striatum | | | 0.000 ± 0.046 | | | | | | 0.024 ± 0.043 | | | | | | -0.101 ± 0.055 | | | | | | -0.050 ± 0.088 | | | | | | 0.153 | | | 0.530 | | | | | 0.818 | | | | |
|  | | | | Thalamus | | | 0.000 ± 0.050 | | | | | | -0.002 ± 0.073 | | | | | | -0.291 ± 0.032 | | | | | | -0.167 ± 0.111 | | | | | | **0.005*** | | | 0.413 | | | | | 0.396 | | | | |
| *ANGPT2* | | | | GM | | | 0.000 ± 0.036 | | | | | | -0.040 ± 0.053 | | | | | | -0.086 ± 0.078 | | | | | | -0.215 ± 0.073 | | | | | | 0.066 | | | 0.224 | | | | | 0.514 | | | | |
|  | | | | WM | | | 0.000 ± 0.097 | | | | | | -0.399 ± 0.212 | | | | | | -0.103 ± 0.064 | | | | | | -0.264 ± 0.054 | | | | | | 0.914 | | | 0.073 | | | | | 0.425 | | | | |
|  | | | | Hipp | | | 0.000 ± 0.039 | | | | | | -0.099 ± 0.092 | | | | | | -0.131 ± 0.048 | | | | | | -0.150 ± 0.062 | | | | | | 0.180 | | | 0.375 | | | | | 0.544 | | | | |
|  | | | | Striatum | | | 0.000 ± 0.052 | | | | | | 0.111 ± 0.083 | | | | | | -0.188 ± 0.134 | | | | | | -0.129 ± 0.084 | | | | | | 0.051 | | | 0.420 | | | | | 0.804 | | | | |
|  | | | | Thalamus | | | 0.000 ± 0.044 | | | | | | 0.007 ± 0.079 | | | | | | -0.257 ± 0.078 | | | | | | -0.390 ± 0.111 | | | | | | **0.001*** | | | 0.480 | | | | | 0.436 | | | | |
| *MEIS1* | | | | GM | | | 0.000 ± 0.092 | | | | | | -0.080 ± 0.102 | | | | | | -0.141 ± 0.086 | | | | | | -0.077 ± 0.050 | | | | | | 0.464 | | | 0.929 | | | | | 0.447 | | | | |
|  | | | | WM | | | 0.000 ± 0.083 | | | | | | -0.006 ± 0.054 | | | | | | 0.052 ± 0.087 | | | | | | 0.032 ± 0.088 | | | | | | 0.604 | | | 0.876 | | | | | 0.933 | | | | |
|  | | | | Hipp | | | 0.000 ± 0.052 | | | | | | 0.049 ± 0.087 | | | | | | -0.074 ± 0.039 | | | | | | -0.097 ± 0.093 | | | | | | 0.130 | | | 0.851 | | | | | 0.609 | | | | |
|  | | | | Striatum | | | 0.000 ± 0.065 | | | | | | 0.049 ± 0.042 | | | | | | 0.082 ± 0.067 | | | | | | 0.147 ± 0.074 | | | | | | 0.166 | | | 0.372 | | | | | 0.898 | | | | |
|  | | | | Thalamus | | | 0.000 ± 0.116 | | | | | | 0.143 ± 0.075 | | | | | | -0.134 ± 0.073 | | | | | | -0.067 ± 0.091 | | | | | | 0.064 | | | 0.245 | | | | | 0.665 | | | | |
| *CLDN1* | | | | GM | | | 0.000 ± 0.072 | | | | | | -0.082 ± 0.099 | | | | | | 0.069 ± 0.039 | | | | | | 0.060 ± 0.059 | | | | | | 0.195 | | | 0.570 | | | | | 0.643 | | | | |
|  | | | | WM | | | 0.000 ± 0.125 | | | | | | -0.281 ± 0.165 | | | | | | -0.034 ± 0.088 | | | | | | -0.178 ± 0.138 | | | | | | 0.807 | | | 0.147 | | | | | 0.628 | | | | |
|  | | | | Hipp | | | 0.000 ± 0.203 | | | | | | -0.122 ± 0.233 | | | | | | -0.025 ± 0.082 | | | | | | 0.076 ± 0.114 | | | | | | 0.612 | | | 0.952 | | | | | 0.513 | | | | |
|  | | | | Striatum | | | 0.000 ± 0.110 | | | | | | 0.394 ± 0.180 | | | | | | 0.303 ± 0.059 | | | | | | 0.408 ± 0.116 | | | | | | 0.243 | | | 0.072 | | | | | 0.285 | | | | |
|  | | | | Thalamus | | | 0.000 ± 0.222 | | | | | | 0.325 ± 0.149 | | | | | | -0.055 ± 0.115 | | | | | | 0.173 ± 0.197 | | | | | | 0.538 | | | 0.111 | | | | | 0.773 | | | | |
